# Prognostic biomarkers for enhanced risk stratification in extraskeletal myxoid chondrosarcoma: a retrospective cohort study

**DOI:** 10.7717/peerj.21497

**Published:** 2026-07-13

**Authors:** Amphun Chaiboonchoe, Jantappapa Chanthercrob, Romgase Sakamula, Thanaphon Likhityungyuen, Sorranart Muangsomboon, Jomjit Chantharasamee, Francis Lee, Krittawat Suwanpukdee, Pannin Thanapipatsiri, Rapin Phimolsarnti, Apichat Asavamongkolkul, Somponnat Sampattavanich, Chandhanarat Chandhanayingyong

**Affiliations:** 1 Siriraj Center of Research Excellence for Precision Medicine and Systems Pharmacology, Department of Pharmacology, Faculty of Medicine Siriraj Hospital, Mahidol University, Bangkok, Thailand; 2 Department of Pathology, Faculty of Medicine Siriraj Hospital, Mahidol University, Bangkok, Thailand; 3 Division of Medical Oncology, Department of Medicine, Faculty of Medicine Siriraj Hospital, Mahidol University, Bangkok, Thailand; 4 Department of Orthopaedics and Rehabilitation, Yale University, Connecticut, United States of America; 5 Department of Orthopaedic Surgery, Faculty of Medicine Siriraj Hospital, Mahidol University, Bangkok, Thailand

**Keywords:** Extraskeletal myxoid chondrosarcoma, Prognostic biomarkers, LASSO–Coxregression, TempO-Seq, Tumor immune microenvironment

## Abstract

**Background:**

Extraskeletal myxoid chondrosarcoma (EMC) is a rare soft-tissue sarcoma driven by the *EWSR1::NR4A3* fusion. Its unpredictable clinical course and lack of validated molecular prognostic tools complicate patient treatment.

**Methods:**

We performed whole-transcriptome targeted RNA sequencing (TempO-Seq) on archival formalin-fixed paraffin-embedded (FFPE) tumor specimens from 12 molecularly confirmed EMC cases. To explore potential biological markers, the cohort was stratified into good-prognosis (*n* = 6; overall survival (OS) >8 years) and poor-prognosis (*n* = 6; OS < 8 years) groups. We conducted differential expression analysis, gene set enrichment analysis (GSEA), and applied least absolute shrinkage and selection operator (LASSO)–Cox regression with bootstrap validation (1,000 iterations). Candidate genes were cross-validated using 31 external EMC cases across public microarray and NGS datasets. Immune infiltration was estimated computationally and spatially validated *via* multiplex immunofluorescence (mIF) on two representative specimens.

**Results:**

We identified 550 differentially expressed genes and derived a three-gene prognostic candidate (*PXN, TYMS, H1FX*) that stratified patients by overall survival in the training cohort (log-rank *P* = 0.0099), despite internal validation indicating model overfitting. *H1FX* was significantly upregulated in EMC versus other cartilaginous tumors across independent datasets (*P* = 0.001). Immune profiling revealed higher B-cell infiltration in low-risk tumors (*P* = 0.005). Proof-of-concept mIF in two specimens suggested a spatially localized immunosuppressive microenvironment in the high-risk tumor, with increased exhausted CD3^+^CD8^+^PD1^+^ T cells (*P* = 6.4 × 10^−5^), FOXP3^+^ regulatory T cells (*P* = 0.006), and elevated PXN protein expression (*P* = 0.008). All mIF comparisons are within-specimen region of interest (ROI) analyses and should be interpreted as hypothesis-generating.

**Conclusions:**

This integrated transcriptomic and spatial immune characterization identifies *PXN, TYMS,* and *H1FX* as exploratory EMC biomarker candidates, with *PXN* showing the most consistent cross-platform support. Immune microenvironment heterogeneity in EMC warrants further investigation. Given the small cohort and model overfitting, these findings are exploratory. Prospective validation in larger, multi-center cohorts is required.

## Introduction

Extraskeletal myxoid chondrosarcoma (EMC) is a rare subtype of mesenchymal tumor, accounting for fewer than 3% of all soft-tissue sarcomas. It primarily affects adults between 30 and 70 years of age and most commonly arises in the deep soft tissues of the thigh or trunk. Although EMC often follows a relatively indolent course, it is associated with a substantial risk of local recurrence and distant metastasis, particularly to the lungs, complicating long-term disease management (Paioli et al., 2021). Complete surgical resection remains the primary treatment strategy and offers the best chance for long-term survival; however, recurrence and late metastatic progression remain considerable clinical challenges.

Despite its often-indolent histologic appearance, EMC demonstrates a highly variable clinical course. Many patients experience prolonged survival, yet a substantial proportion develop late local recurrence or distant metastasis, sometimes occurring many years or even decades after initial diagnosis. This clinical unpredictability presents a major challenge in patient management, as currently available clinicopathologic parameters provide limited ability to distinguish patients with relatively indolent disease from those at higher risk of progression (McGrory et al., 2001; Oliveira et al., 2000; Stacchiotti et al., 2020a).

A defining molecular feature of EMC is the recurrent chromosomal translocation t(9;22) (q22;q12), which generates the *EWSR1::NR4A3* fusion gene. This fusion functions as a key transcriptional driver of tumorigenesis and represents an important diagnostic biomarker that helps distinguish EMC from other morphologically similar soft-tissue sarcomas. Despite this well-characterized genetic hallmark, reliable prognostic biomarkers to predict clinical outcomes in EMC remain lacking. Current prognostic assessments rely primarily on clinicopathologic factors such as tumor size, histologic grade, and metastatic status, which provide only limited predictive accuracy (Brown, Rakoczy & Pretell-Mazzini, 2022; Fice et al., 2022; Minami et al., 2020).

Soft-tissue sarcomas are generally considered immunologically “cold” tumors, characterized by low tumor mutational burden (TMB) and a relatively immunosuppressive tumor microenvironment. This phenotype is particularly pronounced in translocation-driven sarcomas, including EMC, where tumorigenesis is mediated by a single dominant fusion oncogene that generates relatively few immunogenic neoantigens. Consequently, these tumors have historically demonstrated limited responsiveness to immune checkpoint blockade therapies, reinforcing the perception that sarcomas are poorly immunogenic malignancies (Petitprez et al., 2020; Roulleaux Dugage et al., 2021).

However, emerging transcriptomic and spatial profiling studies indicate that immune heterogeneity exists across sarcoma subtypes, with certain tumors exhibiting distinct immune microenvironmental features that may influence prognosis and therapeutic response. These observations raise the possibility that even fusion-driven sarcomas may harbor clinically relevant immune niches that are not captured by traditional clinicopathologic assessment. Several immunohistochemical markers have been explored in EMC (Giner et al., 2023; Gusho et al., 2022; Lenz et al., 2023; Sugino et al., 2023; Zou et al., 2023); however, their expression patterns vary and often lack sufficient specificity or prognostic value. In contrast, transcriptomic approaches such as RNA sequencing provide a more comprehensive strategy for molecular characterization, enabling simultaneous investigation of gene expression patterns, signaling pathways, and immune microenvironmental features associated with disease biology (Gusho et al., 2022; Merry et al., 2021; Zhang et al., 2021).

Because this sarcoma subtype is exceptionally rare, comprehensive molecular investigations are often limited by small cohort sizes, emphasizing the importance of exploratory studies using well-characterized archival specimens. We therefore hypothesized that transcriptomic profiling could identify candidate molecular biomarkers and immune microenvironmental features associated with clinical outcomes in EMC.

Transcriptomic profiling of rare tumors is often limited by the availability of high-quality fresh-frozen tissue, particularly in archival clinical cohorts. The TempO-Seq platform is specifically designed for targeted whole-transcriptome analysis of formalin-fixed paraffin-embedded (FFPE) specimens. It does not require RNA extraction or intact RNA molecules, making it well-suited for degraded archival material. This technology has been successfully applied to retrospective clinical samples and enables reliable gene expression profiling even from long-stored FFPE tissues. As a result, TempO-Seq provides a practical approach for transcriptomic investigation of rare malignancies where sample availability is inherently limited.

In this study, we performed TempO-Seq whole-transcriptome targeted RNA sequencing on archival formalin-fixed paraffin-embedded (FFPE) tumor specimens from patients with EMC to investigate molecular determinants of clinical outcome. By integrating differential gene expression analysis, pathway enrichment analysis, immune cell deconvolution, and exploratory prognostic modeling, we aimed to identify candidate prognostic biomarkers and characterize immune microenvironmental differences associated with patient survival. In addition, multiplex immunofluorescence spatial profiling was performed in representative tumor samples to provide an exploratory protein-level assessment of key transcriptomic findings. To our knowledge, this study represents one of the first integrated transcriptomic and spatial immune microenvironment analyses of EMC. Given the small cohort size, these findings should be interpreted as descriptive and require validation in larger independent cohorts before clinical translation.

## Methods

### Study design and setting

This retrospective analysis was conducted on archival samples spanning two decades. Between April 21, 2008, and June 19, 2018, tumor specimens diagnosed as extraskeletal myxoid chondrosarcoma (EMC) were collected from the Department of Pathology at Siriraj Hospital, Mahidol University (Bangkok, Thailand) and Columbia University (New York, USA). The study protocol was approved by the Institutional Review Boards of both institutions (Siriraj Protocol no. 107/2561, COA no. Si367/2018; Columbia Protocol ID: 2000021232). Written informed consent was obtained for prospective participants, while the requirement for consent was waived by the Boards for the retrospective cohort.

### Participants and study subjects

During the study period, 22 patients with a diagnosis of EMC were initially identified. After histopathological confirmation according to WHO 2020 criteria and review of medical records, eligible patients were included for clinical data extraction and downstream analysis (WHO Classification of Tumours Editorial Board, 2020; Fletcher et al., 2013). Clinical data collected included age, sex, presentation, tumor site and extent, American Joint Committee on Cancer (AJCC) and Enneking staging, treatment modality, and follow-up. Overall survival (OS) was calculated from the date of diagnosis to the date of the last follow-up.

All available formalin-fixed paraffin-embedded (FFPE) blocks were retrieved for molecular analysis. For these cases, two pathologists independently reviewed three µm hematoxylin and eosin-stained sections to confirm the histological subtype (conventional, solid-cellular, or high-grade EMC). Surgical margins were classified following Enneking, Spanier & Goodman (1980). Multidisciplinary Tumor Board discussions were performed for each case to validate the clinical and pathological correlation. Of the 22 patients, seven were excluded due to insufficient RNA quality or critically low gene expression counts, leaving 15 patients with informative gene expression data. One additional sample was identified as an outlier by principal component analysis (PCA) and hierarchical clustering and was subsequently removed. Of the remaining 14 patients, one had died of an unrelated cause, and one had no available prognostic record; both were excluded from prognostic analysis. The final cohort, therefore, comprised 12 EMC patients with complete molecular and clinical data, who were carried forward for all downstream analyses.

For prognostic analysis, patients were categorized into two groups based on survival outcomes: The good-prognosis group (*n* = 6) consisted of patients who showed no evidence of disease or had an indolent disease course, surviving more than 8 years even after the appearance of minimal metastases. The poor-prognosis group (*n* = 6) included patients with rapidly progressive disease who died of disease (DOD) within 8 years of diagnosis. The 8-year threshold was selected rather than the standard 5-year cutoff to reflect the well-documented prolonged natural history of EMC. This disease is characterized by late-onset recurrences and a propensity for disease-related events to occur beyond 5–7 years from initial diagnosis (Ogura et al., 2012; Paioli et al., 2021). This threshold is further supported by published survival data demonstrating prolonged event distribution in EMC, with median overall survival frequently exceeding 10 years and late disease-related mortality beyond 5 years (Brown, Rakoczy & Pretell-Mazzini, 2022; Stacchiotti et al., 2020b). All deaths in the poor-prognosis group were confirmed to be directly attributable to EMC progression. This classification aimed to clearly distinguish indolent long-term survival patterns from aggressive clinical phenotypes. All statistical analyses were considered exploratory given the limited sample size.

### Fluorescence *in situ* hybridization (FISH)

FISH analysis was conducted to assess chromosomal translocations affecting *EWSR1* on paraffin sections using a dual-color break-apart probe (Abbott Molecular, Des Plaines, IL). This probe detects all translocations involving the *EWSR1* gene on chromosome 22q12. Probe hybridization specificity was verified on normal metaphase preparations and the cutoff for variation on paraffin sections was established at 10%. Paraffin-embedded tissues were cut into 4–5 µm-thick sections onto adhesive-coated slides. The tissue sections were baked overnight at 60 °C before hybridization. The slides were treated with protease treatment using a paraffin pretreatment kit (Abbott Molecular, Des Plaines, IL). FISH was performed using standard methods, and hybridization signals were scored on at least 100 distinct nuclei on DAPI-stained slides. All analyses were carried out on a Nikon Eclipse 600 microscope attached to a Cytovision imaging analysis workstation. Cells with a translocation, revealing one fused and one or more ‘split’ signals or deviations from the normal two fused signal pattern, were considered positive for *EWSR1* translocation.

### TempO-Seq assay

Gene expression profiling was performed using the Templated Oligo Detection sequencing (TempO-Seq) platform with the Human Whole Transcriptome 2.0 assay (BioSpyder Technologies), which is optimized for transcriptomic analysis of formalin-fixed paraffin-embedded (FFPE) tissues. The assay utilizes a hybridization-ligation chemistry that enables robust transcript quantification from fragmented RNA commonly present in archival FFPE specimens. The TempO-Seq assay for FFPE samples was performed as described in the user manual (BioSpyder Technologies, 2019). Raw sequencing data have been deposited in the NCBI Sequence Read Archive (SRA) under BioProject accession PRJNA1357027.

In brief, an area of interest on a slide-mounted FFPE section was scraped from the slide and deposited directly into BioSpyder 1X FFPE lysis buffer. The sample was overlaid with mineral oil and incubated at 95 °C for five minutes to dissolve the paraffin. FFPE Protease reagent was added to the FFPE lysate and incubated at 37 °C for 30 min. After a quick homogenization step by vortexing, the lysates were used for the TempO-Seq FFPE assay. A two µL aliquot of the processed lysate was then added to a microplate well containing a mix of annealing buffer and detector oligos (DOs) to measure each targeted gene. The DO panels used in this study were designed against the whole human transcriptome. This mixture was exposed to a temperature decrease from 70 °C to 45 °C, followed by overnight incubation at 45 °C. Then, a nuclease mix was added to degrade unbound and incorrectly bound DOs. Finally, the addition of a ligase mixture enabled the ligation of correctly bound DOs into full-length probes. The enzymes were inactivated by a 15-minute incubation at 80 °C, and the resulting ligated probes were amplified in a polymerase chain reaction (PCR) step. To provide an approximate assessment of statistical power for differential expression analysis, power calculations were performed using the RNASeqPower framework. Assuming dispersion values typical of bulk RNA-seq datasets, the sample size used in this study (*n* = 6 per group) provided an estimated power of 0.98 without technical replicates and remained above 0.80 for detecting genes with large effect sizes (fold change ≥2). A Benjamini–Hochberg false discovery rate (FDR) of 0.05 was applied. Because RNASeqPower assumes independent biological replicates and simplified dispersion models, these calculations should be interpreted as approximate and are not intended as definitive power estimates for this exploratory cohort. The human whole transcriptome assay identified 19,683 unique human genes (22,537 probes). Each gene was measured by one or more probes formed by the ligation of a DO pair, as previously described (Yeakley et al., 2017). TempO-Seq probes for whole transcriptome assays were designed to target only protein-coding genes.

Quality control (QC) was conducted using internal assay controls provided by BioClavis. Samples were required to meet predefined QC thresholds. These included a signal-to-noise ratio >20:1, mapped read percentage in positive RNA controls >80%, average reads per probe >250, and >6 million mapped reads in positive RNA controls. All samples included in this study exceeded these thresholds. Specifically, the assay achieved an average of 7,794,021 mapped reads in positive RNA controls, a signal-to-noise ratio of 174:1, 96% reads mapping to positive controls, and 512 average reads per probe.

Technical reproducibility was assessed using replicate positive RNA controls, which demonstrated excellent correlation (*R*^2^ = 0.99). As expected for archival FFPE specimens, some inter-sample variability in read counts was observed. To account for this variability, downstream data processing included upper-quartile normalization and filtering of low-expressed genes prior to subsequent analyses.

### Data processing, quality control, normalization, and feature standardization

The initial dataset comprised 22 pathologically confirmed EMC cases with raw gene expression data for 22,537 probes. To refine the dataset, genes with zero counts across all samples and those with duplicated gene symbols were discarded. Low-expressed genes were subsequently filtered out using established thresholding methods (Källberg, Vidman & Rydén, 2021). During quality control, seven samples with critically low expression counts were excluded, leaving 15 samples with informative transcriptomic data.

To account for variations in sequencing depth across the cohort, the remaining data underwent global normalization. Specifically, upper quartile (UQ) normalization was applied, and raw counts were converted to counts per million (CPM) using the *edgeR* package in R (Robinson, McCarthy & Smyth, 2010). Unsupervised analyses, including principal component analysis (PCA) and hierarchical clustering, were then conducted to assess batch effects and identify potential outliers, leading to the exclusion of one outlier sample. Finally, two samples lacking valid clinical prognostic information (one unrelated death and one missing record) were excluded. This established the final analytical cohort of 12 EMC patients encompassing 9,909 genes for downstream analysis.

Although UQ normalization corrects for between-sample library size differences, penalized regression models such as Least Absolute Shrinkage and Selection Operator (LASSO) are sensitive to differences in predictor variance. Therefore, prior to prognostic model construction, normalized gene expression values were further standardized using *z*-score transformation. This step ensured that all genes were placed on a common mathematical scale (mean = 0, standard deviation = 1). This standardization enabled fair comparison of predictor effects and stable coefficient estimation during LASSO-based feature selection.

### Univariate Cox regression analysis of clinical covariates

To assess the prognostic relevance of clinical characteristics, univariate Cox proportional hazards regression was performed for all estimable clinical variables. These included age, sex, ethnicity, tumor size, *EWSR1* translocation status, cellularity grade, tumor location, and FFPE block age. Two variables, metastasis at diagnosis and surgical margin status, exhibited complete data separation (zero survival events in the reference category). Because standard Cox regression fails to converge under these conditions, Firth’s penalized likelihood Cox regression (*via* the *coxphf* R package) was applied to calculate finite hazard ratios, 95% confidence intervals, and reliable *P* values. Due to the small cohort size (*n* = 12) and the limited number of events, a formal multivariable Cox regression was not performed. This decision was made to avoid model overfitting.

### Construction of the prognostic model

To develop a prognostic model for overall survival, gene expression data were analyzed using Cox proportional hazards regression implemented in the R package survival. Initially, univariate Cox regression was performed for each differentially expressed gene to evaluate its association with overall survival. Genes demonstrating prognostic relevance were subsequently subjected to Least Absolute Shrinkage and Selection Operator (LASSO) regression using the glmnet package (v4.1) to reduce dimensionality and identify the most informative gene set.

LASSO model tuning was performed using leave-one-out cross-validation (LOOCV; *k* = 12) *via* the cv.glmnet function, with the optimal penalty parameter selected based on the minimum cross-validated partial likelihood deviance (*λ*min). The selected genes were then used to construct the final prognostic model using Cox proportional hazards regression. Regression coefficients were used to derive a risk score formula based on standardized gene expression values.

Patients were stratified into high-risk and low-risk groups according to the calculated risk scores. Differences in overall survival between groups were evaluated using Kaplan–Meier survival analysis and the log-rank test, with statistical significance defined as *P*  <  0.05.

### Internal validation and statistical considerations for small sample size

Given the limited cohort size (*n* = 12), several methodological safeguards were implemented to minimize the risk of model overfitting and improve the reliability of prognostic estimates. Feature selection and coefficient shrinkage were performed using least absolute shrinkage and selection operator (LASSO) penalized Cox regression, an approach well suited for high-dimensional transcriptomic data in which the number of predictors exceeds the number of samples. Model training employed leave-one-out cross-validation (LOOCV), allowing each sample to contribute to both model development and validation while maximizing the available training data.

Internal validation of the prognostic model was performed using bootstrap resampling to estimate the optimism-corrected concordance index (C-index), following the framework described by Harrell Jr., Lee & Mark (1996). A total of 1,000 bootstrap iterations were conducted. In each iteration, a bootstrap sample (sampling with replacement, *n* = 12) was drawn from the original dataset, and the LOOCV LASSO–Cox model was refitted. Model performance was assessed by calculating the C-index on the bootstrap sample (apparent performance) and on the original dataset (test performance). Optimism was defined as the difference between these two values. The mean optimism across all iterations was subtracted from the apparent C-index of the original model to obtain the optimism-corrected C-index.

Model calibration was evaluated using bootstrap calibration curves generated at the median follow-up time using the rms package, which assessed the agreement between predicted and observed survival probabilities.

Together, the combination of penalized regression, cross-validation, and bootstrap optimism correction provides a robust framework for model development and internal validation in small exploratory cohorts. Nevertheless, external validation in independent datasets will be necessary to confirm the generalizability of the proposed prognostic model.

### Immune cell infiltration and deconvolution analysis

To comprehensively characterize the tumor immune microenvironment (TME) of extraskeletal myxoid chondrosarcoma (EMC), immune cell infiltration was estimated from bulk RNA sequencing data using six orthogonal deconvolution algorithms available through the TIMER2.0 platform: TIMER, CIBERSORT, CIBERSORT-ABS, xCell, EPIC, and MCP-counter (Aran, Hu & Butte, 2017; Becht et al., 2016; Chen et al., 2018; Li et al., 2020b; Newman et al., 2015; Racle et al., 2017). To enable standardized cross-algorithm comparisons, the high-resolution cell subtypes reported by specific algorithms (*e.g.*, the 22 subpopulations from CIBERSORT) were logically aggregated into six canonical parent immune lineages these comprised B cells, CD4^+^ T cells, CD8^+^ T cells, Macrophages, Neutrophils, and Myeloid dendritic cells. Non-immune stromal subsets and composite microenvironment scores were excluded from this specific analysis.

For statistical comparison, the cohort (*n* = 12) was stratified into high-risk (*n* = 6) and low-risk (*n* = 6) subgroups based on the LASSO–Cox risk score. Differences in the estimated immune cell abundances between the two risk groups were evaluated using the non-parametric two-sided Wilcoxon rank-sum test. The magnitude of these differences was quantified using the rank-biserial correlation coefficient (*r*) to determine effect size. To control for false positives across the 36 simultaneous comparisons (6 lineages × 6 algorithms), raw *P* values were adjusted using the Benjamini–Hochberg False Discovery Rate (FDR) procedure. Statistical analyses and data visualization, including jitter-overlaid boxplots, were conducted in R using the *ggplot2* and *ggpubr* packages.

### Gene set enrichment analysis

To characterize immune-related and oncological pathway activity in EMC, gene set enrichment analysis (GSEA) was performed using the *fgsea* package (v1.24.0) (Korotkevich et al., 2016) with 10,000 permutations. Genes were ranked by log2 fold change between poor and good-prognosis groups derived from the differential expression analysis. Hallmark gene sets (*n* = 50) from the Molecular Signatures Database (MSigDB v7.5.1) (Liberzon et al., 2011) were used as reference gene sets. The standard GSEA scoring type was applied (scoreType = “std”). A normalized enrichment score (NES) threshold of |NES| > 1.0 with FDR < 0.05 was used as a lenient exploratory threshold appropriate for small-sample studies.

### Cross-study comparison and external cohort evaluation

To contextualize the identified prognostic candidates within the existing extraskeletal myxoid chondrosarcoma (EMC) transcriptomic literature, we reviewed prior molecular profiling efforts. Early studies by Sjögren et al. (2003) and Subramanian et al. (2005) utilized cDNA microarrays; however, these datasets were generated prior to routine public deposition and are unavailable for reanalysis. While Davis et al. (2017) and Möller et al. (2011) also conducted molecular profiling of EMC, whole-transcriptome expression matrices were not readily accessible in public repositories (Davis et al., 2017; Möller et al., 2011; Sjögren et al., 2003; Subramanian et al., 2005).

Through a formal request to the journal editors, we obtained both the whole-transcriptome expression matrices and the matched clinical data for the six EMC cases profiled using the MI-ONCOSEQ platform by Davis et al. (2017). Although overall survival data were available for this cohort, the cases exhibited profound clinical heterogeneity, including diverse biopsy sites (*e.g.*, primary tumors, pulmonary metastases, and gluteal metastases) and highly varied histories of systemic therapies and radiation. Given these severe clinical confounders and the extremely limited sample size (*n* = 6), evaluating prognostic outcomes in this subset was deemed statistically inappropriate. Consequently, this dataset was utilized strictly to test the baseline cross-platform reproducibility of our candidate genes.

To assess lineage specificity, we performed cross-study analyses using two independent, publicly available microarray datasets from the Gene Expression Omnibus (GEO) that lacked matched survival annotations. The first dataset, GSE24369, contains transcriptomic profiles comparing EMC tumors (*n* = 6) against other soft-tissue sarcoma (STS) subtypes (*n* = 36). The second dataset, GSE6481, directly compares EMC tumors (*n* = 19) against a broad background of other chondrosarcomas (*n* = 128).

Expression levels for the three LASSO-derived prognostic candidate genes (*PXN, TYMS*, and *H1FX*) were extracted and analyzed across these three external cohorts. For the GEO datasets, differential expression was evaluated between the EMC and non-EMC groups using the two-tailed Wilcoxon rank-sum test, with effect sizes reported as log2 fold-change (log_2_FC). To account for baseline technical differences between our internal TempO-Seq platform (log_2_CPM; *n* = 12) and the external MI-ONCOSEQ platform (Davis et al., log_2_[RPKM + 1]; *n* = 6), we applied a within-dataset *z*-score transformation to standardize the expression values prior to comparison. Statistical differences in the normalized expression distributions between these two independent platforms were evaluated using a two-sided Mann–Whitney *U* test.

Collectively, while the lack of baseline survival annotations in the GEO cohorts and the significant clinical confounding factors in the Davis et al. dataset precluded direct validation of clinical outcomes, these cross-study evaluations allow us to rigorously establish the cross-platform reproducibility of baseline gene expression and the lineage specificity of our exploratory panel. Statistical analyses and visualizations were performed in R (v4.3.1) using the *ggplot2* and *ggpubr* packages.

### Single-plex and multiplex immunofluorescence staining

Immunofluorescence staining comprised two panels: single-plex immunofluorescence for biomarker protein expression quantification (PXN, TYMS, and H1FX) and multiplex immunofluorescence (mIF) for spatial immune cell phenotyping. Multiplex tissue-based cyclic immunofluorescence (t-CyCIF) was performed according to a previously described protocol (Lin et al., 2018).

### Tissue preparation

Slides were dewaxed and rehydrated using standard protocols, followed by antigen retrieval in citrate buffer (pH 6.0, Dako Omnis) for 20 min at 95 °C.

### Single-plex immunofluorescence

Single-plex staining was performed using the following unconjugated primary antibodies: anti-Paxillin [Y113] (Abcam, ab32084), anti-TYMS (Sigma-Aldrich, HPA074922), and anti-H1-10 (Sigma-Aldrich, HPA068431). Primary antibodies were incubated overnight at 4 °C, followed by incubation with a fluorophore-conjugated secondary antibody (Invitrogen, A-31573) for 1 h at room temperature. Nuclei staining was performed with Hoechst 33342, followed by imaging using the PhenoImager Fusion system (Akoya Biosciences, Marlborough, MA, USA). Protein expression was quantified as mean fluorescence intensity per cell across multiple regions of interest (ROIs).

### Multiplex immunofluorescence (mIF)

mIF staining was performed using conjugated primary antibodies across two sequential staining cycles. Cycle 1 comprised: anti-CD8a [AMC908] (eBioscience, 53-0008-82), anti-PD1 [EPR4877(2)] (Abcam, ab201825), and anti-CD3d [EP4426] (Abcam, ab208514). Cycle 2 comprised: anti-CD4 [EPR6855] (Abcam, ab196372), anti-CD20 [L26] (eBioscience, 50-0202-82), and anti-FOXP3 [236A/E7] (eBioscience, 41-4777-82). For each cycle, three conjugated antibodies were incubated overnight at 4 °C, followed by nuclear staining with Hoechst 33342 and imaging using the CyteFinder II HT system (RareCyte Inc., Seattle, WA, USA). Following completion of imaging for each cycle, conjugated fluorophores were chemically inactivated by photobleaching prior to proceeding to the next cycle. Upon completion of all cycles, slides were stained with hematoxylin and eosin (Abcam, ab245880) for morphological reference.

### Image processing and cell classification

Multi-cycle fluorescence images were registered and stitched using ASHLAR (https://github.com/labsyspharm/ashlar). Nuclear segmentation was performed using StarDist (https://github.com/stardist/stardist). Cell classification and immune phenotyping were performed in QuPath software using threshold-based multi-marker co-expression criteria. Immune cell populations were defined as follows: B cells (CD20^+^), helper T cells (CD3^+^CD4^+^), cytotoxic T cells (CD3^+^CD8^+^), regulatory T cells/Tregs (CD3^+^CD4^+^FOXP3^+^), exhausted helper T cells (CD3^+^CD4^+^PD1^+^), and exhausted cytotoxic T cells (CD3^+^CD8^+^PD1^+^). PD1 positivity was not used as a standalone classification criterion; all PD1^+^ populations were strictly co-classified with definitive T-cell lineage markers (CD3, CD4, or CD8). Data were expressed as mean immune cell density (cells/mm^2^) for mIF and mean fluorescence intensity per cell for single-plex staining.

### Statistical analysis of immunofluorescence data

ROI-level comparisons of single-plex fluorescence intensity between the two representative specimens (one poor-prognosis and one good-prognosis case) were performed using the Wilcoxon rank-sum test (*n* = 5 ROIs per specimen). ROI-level immune cell density comparisons between transcriptomic risk groups were similarly evaluated using the Wilcoxon rank-sum test (*n* = 10 ROIs per specimen). Because ROIs represent technical replicates within individual tumor specimens rather than independent biological samples, all immunofluorescence results are presented as descriptive and hypothesis-generating observations. *P* values are reported for completeness but must not be interpreted as evidence of biological group-level differences.

## Results

### Patient and tumor characteristics

**Figure 1:**
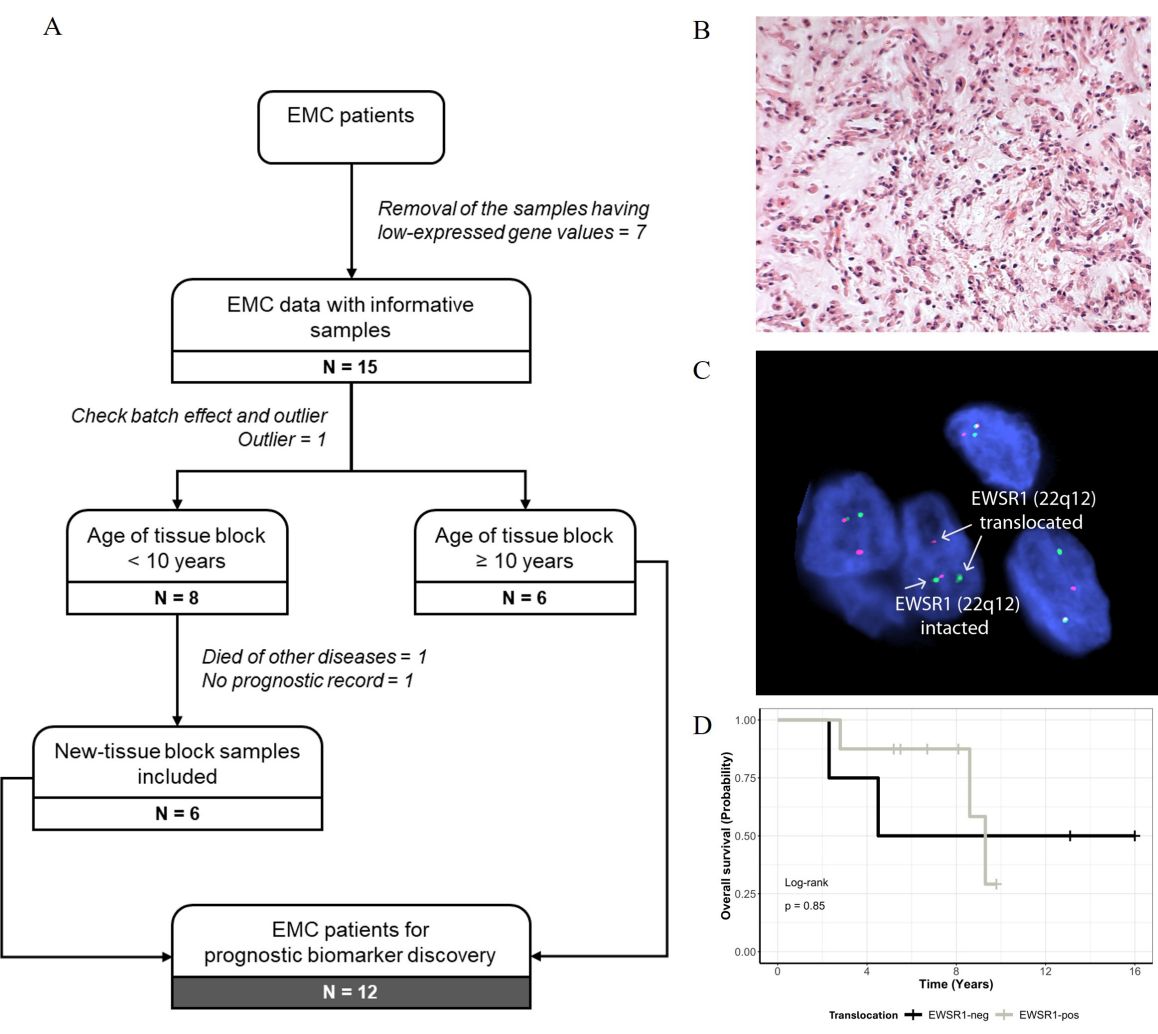
Histopathological features and *EWSR1* translocation status in extraskeletal myxoid chondrosarcoma. (A) Patient selection criteria. (B) The classic EMC displays a lobulated architecture and a reticular growth pattern. The tumor cells exhibit uniformity, with ovoid-to-spindled nuclei and eosinophilic cytoplasm, as shown in hematoxylin and eosin (H&E) stained. (C) FISH demonstrates rearrangement of *EWSR1* (22q12). (D) Overall survival comparison between the *EWSR1*+ and *EWSR1*− group (*P* = 0.85). Abbreviations: EMC, extraskeletal myxoid chondrosarcoma; *EWSR1*, EWS RNA-binding protein 1; FISH, fluorescence *in situ* hybridization; H&E, hematoxylin and eosin.

A total of 12 EMC patients met all inclusion and molecular quality control criteria and were included in the final analysis, as described in the Methods and illustrated in Fig. 1A. This study aimed to identify molecular features associated with clinical outcomes. Of the 12 EMC patients, seven (60%) were women and five (40%) were men (male-to-female ratio, 0.71:1). The median age at diagnosis was 51.5 years (range, 37–77 years). The median follow-up for the entire cohort was 88.8 months (range 27.6–192 months). In eight out of 12 EMC patients, *EWSR1* rearrangements were detected by FISH. The *EWSR1* fusion gene in EMC does not reliably predict overall survival (log-rank *P* = 0.85), confirming its role as a diagnostic rather than prognostic marker. Patients were classified into good-prognosis (*n* = 6; OS > 8 years, no notable metastasis) and poor-prognosis (*n* = 6; progressive disease or death from disease, OS < 8 years) groups. The 8-year threshold reflects the prolonged natural history of EMC, in which clinically meaningful events frequently occur beyond 5–7 years. Clinical characteristics are summarized in Table 1. All findings should be interpreted in the context of a small cohort and require validation in independent datasets.

**Table 1:** Clinical and pathological characteristics of clinical data of extraskeletal myxoid chondrosarcoma patients.

Clinical characteristics	*n* = 12
Age at diagnosis (years)	
Median (range)	51.5 (37–77)
Gender	
Male	5
Female	7
M:F ratio	0.71:1
Location	
Thigh	7
Leg & foot	2
Buttock & trunk	2
Upper extremity	1
Size	
<5 cm	3
5–10	1
>10 cm	8
Histology	
Classical	9
High grade solid-cellular	1
High grade spindle	2
Fusion gene (FISH)	
*EWSR1-NR4A3* (*EWSR1*+)	8
No *EWSR1* rearrangement (*EWSR1*−)	4
Margins	
R0	4
R1	2
R2	6
Prognosis	
No metastasis/Prolonged life >8 years after metastasis (Good)	6
Death shortly after metastasis (Poor)	6
Follow-up (months)	
NED	3
AWD	4
DOD	5

**Abbreviations:**

AWD alive with disease DOD died of disease EWSR1 EWS RNA-binding protein 1 FISH fluorescence *in situ* hybridization M:F male-to-female NED no evidence of disease NR4A3 nuclear receptor subfamily 4 group A member 3

### Analysis of differentially expressed genes and gene set enrichment analysis of EMC between good *vs* poor prognosis

**Figure 2:**
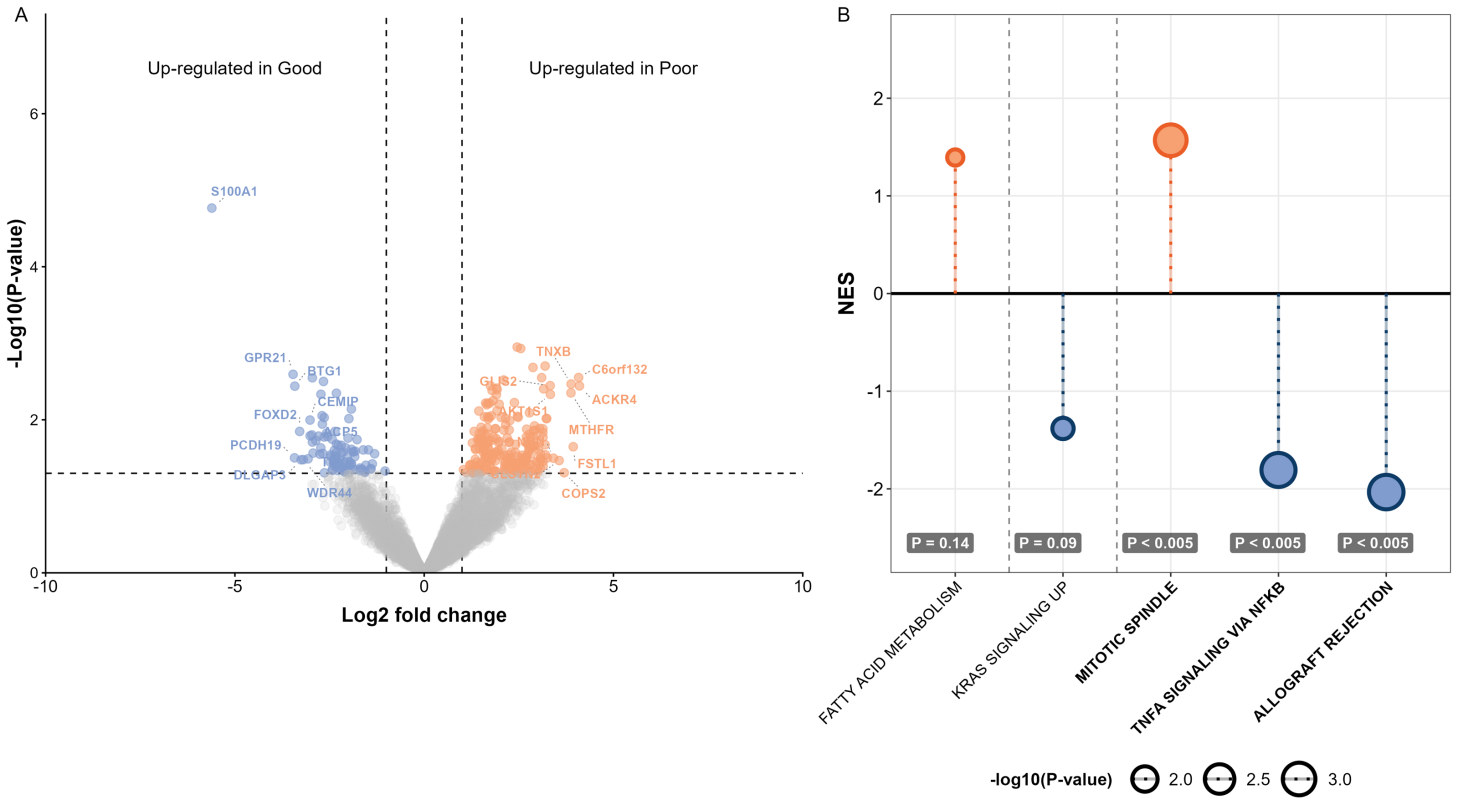
Differential gene expression analysis in extraskeletal myxoid chondrosarcoma. (A) Volcano plot comparing good *vs* poor prognosis. Differentially expressed genes meeting significance thresholds are highlighted in the upper-left and upper-right rectangles. Log_2_-fold changes in expression *versus* −log_10_ (*P* value) are shown on the *x*- and *y*-axis, respectively. (B) GSEA of top enriched pathways in good *vs* poor prognosis, normalized enrichment score (NES) across all Hallmark gene sets, with statistically significant pathways indicated. Abbreviations: EMC, extraskeletal myxoid chondrosarcoma; GSEA, gene set enrichment analysis; NES, normalized enrichment score.

To characterize the molecular differences between the good- and poor-prognosis groups, we performed TempO-Seq whole-transcriptome targeted RNA sequencing in the final 12 EMC patients. After normalizing gene expression data and correcting for batch effects (Fig. S1), we identified differentially expressed genes between the two groups, yielding 550 (467 upregulated and 83 downregulated; |log FC| > 1 and *P* < 0.05), as shown in the volcano plot (Fig. 2A). *S100A1, KCNC3, PCDH19, GPR21,* and *BTG1* were the most highly expressed genes in the good-prognosis group, whereas *SMARCD3, TMEM184A, SNX33, NOC2L,* and *DENND2A* were enriched in the poor-prognosis group. *S100A1* encodes an S100-family protein involved in cell-cycle progression and differentiation; in ovarian cancer, its expression correlates with tumor grade and lymph node metastasis, suggesting a role in disease progression.

To explore functional differences between poor- and good-prognosis groups, we performed a GSEA using 10,000 permutations against Molecular Signatures Database hallmark gene sets (*n* = 50), with genes ranked by log_2_FC. The three hallmarks with the most extreme NESs and the lowest *P* values were allograft rejection (*MMP9, HLA-DRA, FGR*), TNF-α signaling *via* NF-κB (*BTG1, EGR2, PTGER4*); upregulated in the good-prognosis group. The third hallmark was mitotic spindle (*PXN, CENPJ, KATNA1*; upregulated in the poor-prognosis group; Fig. 2B). Results for all immune-related and oncological hallmark pathways are shown in Fig. S2 (immune-focused lollipop plot).

While fatty acid metabolism (normalized enrichment score (NES) = 1.39) and *KRAS* Signaling Up (NES = −1.38) showed trends toward enrichment in the poor- and good-prognosis groups, respectively, these did not reach the significance threshold (FDR > 0.05).

Gene set enrichment analysis (GSEA) revealed distinct immune-related and oncological pathways associated with EMC prognosis. In the poor-prognosis group, apoptosis was the most significantly enriched pathway (*P* < 0.05). Additional pathways with positive enrichment trends in this group included myogenesis, bile acid metabolism, coagulation, and interferon-α response.

In contrast, the good-prognosis group was characterized by the significant enrichment of IL2–STAT5 signaling (*P* < 0.005). In this context, IL2–STAT5 signaling likely reflects active and beneficial immune engagement, such as effector T-cell activity. Furthermore, the good-prognosis group showed consistent enrichment trends across multiple other immune and inflammatory pathways, including interferon-*γ* response, complement, inflammatory response, IL6–JAK–STAT3 signaling, TNF-α signaling *via* NF-*κ*B, and allograft rejection. Individual *P* values for these additional immune hallmarks did not reach conventional statistical significance, a finding expected given the small cohort size (*n* = 6 per group). Nevertheless, the consistent directional enrichment of immune-activation pathways suggests that enhanced immune responses are associated with favorable EMC outcomes.

Taken together, the GSEA results indicate that poor-prognosis EMC is characterized by apoptosis pathway activation and specific metabolic or coagulation trends. Conversely, good-prognosis tumors show statistically significant IL2–STAT5 signaling alongside a broader enrichment in pathways associated with adaptive immune engagement and inflammation, including interferon-*γ* signaling, complement activation, and allograft rejection. These pathway-level observations provide biological context for the immune cell infiltration findings described in the following section.

### LASSO analysis identifies candidate prognostic genes

To evaluate the prognostic impact of standard clinical variables, we performed univariate proportional hazards regression on age, sex, ethnicity, tumor size, location, cellularity, *EWSR1* fusion status, metastasis at diagnosis, surgical margin status, and FFPE block age. For covariates exhibiting complete separation (zero survival events in the reference group, specifically surgical margins and metastasis), Firth’s penalized Cox regression was utilized to compute finite hazard ratios. As summarized in Table S1, positive surgical margins (R1/R2) were associated with decreased overall survival (HR = 14.13, 95% CI [4.06–Inf], *P* = 0.021). Conversely, other clinical variables, including age, tumor size, and metastasis at diagnosis, did not reach statistical significance (*P* > 0.05), likely due to the limited statistical power of the small sample size. Due to the limited number of events (*n* = 5), a formal multivariable Cox regression incorporating these clinical covariates alongside the transcriptomic data was not performed to avoid severe model overfitting.

Given the absence of robust clinical predictors, we performed a univariate Cox regression analysis on the 550 differentially expressed genes. The analysis of the good- and poor-prognosis groups identified 14 genes potentially associated with EMC prognosis (*P* < 0.05; Table S2). *ZSCAN21, TYMS, PXN, TRIM11,* and *H1FX* were the top five genes with the highest HR (all >2). Higher expression of these genes was associated with reduced survival for EMC patients. Conversely, genes with an HR < 1, such as *COL12A1*, were associated with improved survival.

To explore potential prognostic panels and mitigate dimensionality in our small cohort, LASSO–Cox regression was used to select the most predictive features from the 14 candidate genes. Following *z*-score normalization of expression data, the LASSO algorithm identified a three-gene candidate panel comprising *PXN*, *TYMS*, and *H1FX* (Figs. S3A and S3B). Individual Kaplan–Meier survival analyses confirmed that higher expression of each of these three selected genes is associated with worse overall survival (Fig. S3C).

To evaluate the combined prognostic value of these candidate genes, they were incorporated into a multivariable Cox proportional hazards regression to derive their final predictive model weights. The risk score was calculated using these derived coefficients according to the following formula:

\begin{eqnarray*}\text{Risk Score}& =& (1.16\times PXN~\mathrm{expression})+(1.58\times H1FX~\mathrm{expression})\nonumber\\\displaystyle & & +\,(1.0\times TYMS~\mathrm{expression}). \end{eqnarray*}

**Figure 3:**
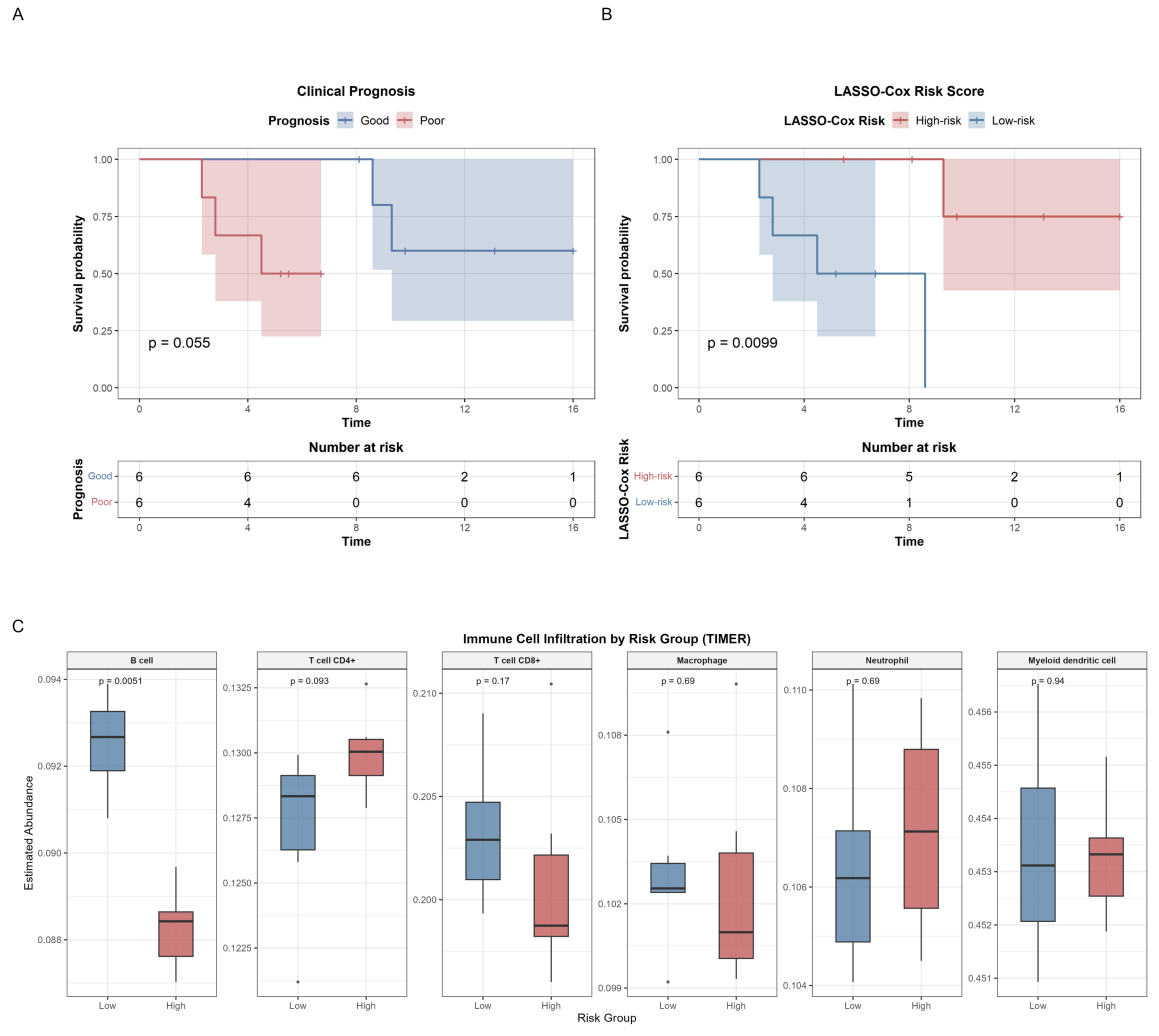
Three-gene prognostic candidate (*H1FX, PXN*, and *TYMS*) and immune cell infiltration in extraskeletal myxoid chondrosarcoma. (A) The overall survival between the good- and poor-prognosis groups (*P* = 0.055). (B) The overall survival between the high- and low-risk groups (*P* = 0.0099). Training cohort, apparent performance only; optimism-corrected C-index 0.10; this plot reflects in-sample separation and should not be interpreted as evidence of generalizable prognostic discrimination. (C) Estimated abundances of immune cell infiltrates in high-risk and low-risk EMC groups, calculated using the TIMER algorithm. Abbreviations: EMC, extraskeletal myxoid chondrosarcoma; *H1FX*, H1 histone family member X; *PXN*, paxillin; TIMER, Tumor Immune Estimation Resource; *TYMS*, thymidylate synthase.

Based on the initial clinical classification, Kaplan–Meier survival analysis showed that patients in the clinically defined poor-prognosis group exhibited a trend toward worse overall survival compared with the good-prognosis group; however, this difference did not reach statistical significance (log-rank *P* = 0.055; Fig. 3A).

To further refine prognostic stratification, a transcriptomic risk score was calculated using the three-gene LASSO–Cox model comprising *PXN, TYMS*, and *H1FX*. Patients were subsequently stratified into high- and low-risk groups based on this risk score. Kaplan–Meier survival analysis demonstrated that this gene-expression-based model effectively separated patients within this small training cohort by overall survival outcome, with the high-risk group showing markedly poorer survival (log-rank *P* = 0.0099; Fig. 3B).

Importantly, the gene-expression-based risk classification was not identical to the original clinical prognosis grouping used for differential expression analysis, although there is partial overlap between the two classifications. This difference reflects the independent derivation of the transcriptomic risk model, indicating that the molecular panel captures additional biological variation beyond the initial clinical grouping.

However, when a multivariable Cox regression model was constructed using this three-gene transcriptomic panel (*PXN, TYMS, H1FX*), the limitations of the cohort size became apparent. Given the small cohort size (*n* = 12) relative to the number of events (*n* = 5), the model demonstrated perfect apparent discrimination on the training data, yielding an apparent Concordance index (C-index) of 1.00. To quantify the degree of overfitting and evaluate model stability, rigorous internal validation was performed using 1,000 bootstrap resamples. The bootstrap analysis revealed an extreme mean optimism of 0.9013. Consequently, the optimism-corrected C-index dropped to 0.10. Model calibration was also assessed using bootstrap calibration plots generated at 1, 2, 3, 4, and 5 years (Fig. S5). Predicted survival probabilities were highly polarized, consistent with severe model overfitting; however, the directional separation between low-risk and high-risk groups remained consistent across all time points (Fig. S5). These validation metrics confirm that while the transcriptomic panel achieves complete separation in this specific training cohort, the multivariable model is heavily overfitted. This is a known mathematical limitation when applying multivariable regression to ultra-rare tumor datasets with limited events.

Consequently, while the individual biomarkers (*PXN, TYMS, H1FX*) suggest strong prognostic associations in EMC, the combined risk model lacks the generalizable stability required for clinical application. These findings should therefore be interpreted strictly as an exploratory, hypothesis-generating framework that identifies biological targets for future validation in larger, multicenter cohorts.

### *In silico* immune cell deconvolution

To characterize the immune microenvironment of EMC and its relationship with clinical outcomes, we performed an *in-silico* deconvolution of bulk RNA sequencing data using six independent algorithms (TIMER, CIBERSORT, CIBERSORT-ABS, xCell, EPIC, and MCP-counter) *via* the TIMER2.0 platform. This approach estimated the abundance of six major immune lineages: B cells, CD4^+^ T cells, CD8^+^ T cells, macrophages, neutrophils, and myeloid dendritic cells (Fig. S4).

Across the multi-algorithm ensemble, B cells emerged as the most consistent prognostic indicator. Higher estimated B-cell abundance was significantly associated with the low-risk group as determined by TIMER (*P* = 0.005), CIBERSORT-ABS (*P* = 0.020), and MCP-counter (*P* = 0.006). While these associations reached nominal significance (*P* < 0.05), they did not remain significant after stringent Benjamini–Hochberg FDR correction, likely due to the limited sample size (*n* = 12).

In contrast, other immune populations did not exhibit consistent significant differences across the algorithms. For instance, while some methods suggested trends for CD4^+^ T cells and macrophages, the results were either non-significant or inconsistent between platforms. These data suggest that a B-cell-enriched microenvironment is a distinct feature of low-risk EMC tumors.

### Cross-cohort analysis and lineage specificity of prognostic biomarkers

To assess the cross-platform reproducibility of our 3-gene candidate and evaluate its lineage specificity, we analyzed three independent, publicly available datasets (Fig. S6).

In the GSE24369 dataset, which compares the transcriptomic profiles of six EMC tumors against 36 other soft-tissue sarcomas (Fig. S6A), the signature genes suggested stable, uniform baseline expression. Expression did not differ between EMC and other sarcomas (*PXN*: log_2_FC = −0.02, *P* = 0.793; *TYMS*: log_2_FC = 0.02, *P* = 0.739; *H1FX*: log_2_FC = −0.26, *P* = 0.129). Conversely, evaluating the GSE6481 dataset, which compares 19 EMC tumors against 128 other cartilaginous tumors/chondrosarcomas (Fig. S6B) revealed distinct lineage-specific expression patterns. Crucially, *H1FX* was statistically significantly upregulated in EMC compared with other chondrosarcomas (log_2_FC = 0.64, *P* = 0.001). In contrast, *PXN* and *TYMS* showed non-significant trends toward lower expression in EMC relative to other chondrosarcomas (*PXN*: log_2_FC = −0.52, *P* = 0.061; *TYMS*: log_2_FC = −0.44, *P* = 0.085).

To further account for baseline technical differences and evaluate external consistency, we compared our internal TempO-Seq platform (Log_2_CPM; *n* = 12) against an external MI-ONCOSEQ platform dataset (Davis et al., Log_2_[RPKM + 1]; *n* = 6) by applying a within-dataset *z*-score transformation. Statistical evaluation using a two-sided Mann–Whitney U test demonstrated robust cross-platform consistency for all three biomarkers. As shown in Fig. S6C, the normalized expression distributions of *PXN* (*P* = 0.963), *H1FX* (*P* = 0.888), and *TYMS* (*P* = 1) did not significantly differ between our internal cohort and the external cohort. This confirms that our normalization strategy successfully bridges distinct sequencing platforms, validating the baseline comparability of these genes in an independent dataset.

### Proof-of-concept immunofluorescence assessment

Given limited tissue availability, immunofluorescence staining was performed on two representative FFPE specimens (one poor-prognosis and one good-prognosis case) as proof-of-concept validation. Consequently, this analysis should be considered illustrative orthogonal evidence rather than formal validation and does not permit statistical comparisons across the full cohort. All IF findings are therefore presented as descriptive and hypothesis-generating. Regions of interest (ROI) measurements represent technical replicates within each tumor specimen and should not be interpreted as independent biological samples or evidence of cohort-level differences. These observations provide qualitative spatial context for transcriptomic findings but require validation in larger cohorts.

### Single-plex IF: prognostic protein expression

**Figure 4:**
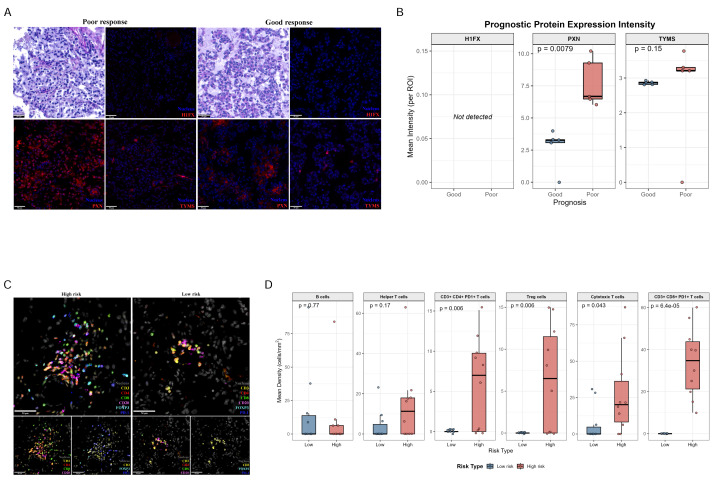
Immunofluorescence analysis of candidate biomarkers and the immune microenvironment in representative extraskeletal myxoid chondrosarcoma tumors. (A) Representative hematoxylin and eosin (H&E) staining and single-plex immunofluorescence images illustrating the expression of H1FX, PXN and TYMS in two representative EMC tumors: one poor-prognosis case and one good-prognosis case. Nuclei are counterstained with DAPI. (B) Quantification of H1FX, PXN and TYMS fluorescence intensity across multiple regions of interest (ROIs) within each specimen. PXN showed higher signal intensity in ROIs from the poor-prognosis tumor, whereas TYMS displayed similar signal levels across both tumors. H1FX protein signal was not detectable under the assay conditions. (C) Representative multiplex immunofluorescence images illustrating immune cell composition in tumors classified as transcriptomic high-risk and low-risk. Markers shown include CD3, CD4, CD8, CD20, FOXP3, and PD1. (D) ROI-level quantification of immune cell densities (cells/mm^2^) within the analyzed specimens. Because ROIs represent technical replicates from individual tumors rather than independent biological samples, results are presented descriptively without formal statistical comparison between groups. Scale bars: 50 µm. Abbreviations: DAPI, 4′,6-diamidino-2-phenylindole; EMC, extraskeletal myxoid chondrosarcoma; H&E, hematoxylin and eosin; H1FX, H1 histone family member X; IF, immunofluorescence; mIF, multiplex immunofluorescence; PD1, programmed cell death protein1; PXN, paxillin; ROI, region of interest; TYMS, thymidylate synthase. Important note: All immunofluorescence data derive from two individual representative specimens only (one poor-prognosis and one good-prognosis case). *P* values shown in Fig. 4B reflect Wilcoxon rank-sum comparisons between ROIs (*n* = 5 ROIs per specimen) of these two individual cases and represent within-specimen regional variation only; they cannot be interpreted as biological group-level statistical evidence. Similarly, *P* values shown in Fig. 4D reflect ROI-level comparisons (*n* = 10 ROIs per specimen) between the same two representative specimens and must not be interpreted as cohort-level statistical differences. No formal statistical inference between patient groups is intended or possible from these two-sample immunofluorescence data.

PXN protein expression was statistically significantly higher in the poor-prognosis (high-risk) sample across five ROIs (mean intensity: 7.73 *vs* 2.73; Wilcoxon rank-sum test on ROI-level measurements, *P* = 0.0079). This finding provides protein-level support for the transcriptomic data. TYMS showed a non-significant numerical trend toward higher expression in the poor-prognosis sample (mean intensity: 3.20 *vs* 2.86; *P* = 0.15). H1FX protein was not detected in either sample (mean intensity = 0.00 in both), suggesting potential RNA–protein discordance that may reflect technical limitations of FFPE-based IF for chromatin-associated nuclear proteins (Fig. 4B).

### Multiplex IF: immune microenvironment

mIF quantified six immune cell phenotypes across 10 ROIs per sample to evaluate the tumor immune microenvironment. As shown in Fig. 4D, bulk transcriptomics identified global B-cell enrichment in low-risk tumors. However, mIF ROI analysis showed no statistically significant difference in B-cell density (*P* = 0.77) or helper T-cell density (*P* = 0.17) between the risk groups. In contrast, mIF revealed marked differences in T-cell functional states that bulk transcriptomic deconvolution could not resolve. Total cytotoxic T cells were significantly enriched in the high-risk sample (*P* = 0.043), as were regulatory T cells (Tregs; *P* = 0.006). When assessing markers of immune exhaustion, both CD3^+^CD4^+^PD1^+^ T cells (*P* = 0.006) and CD3^+^CD8^+^PD1^+^ cytotoxic T cells (*P* = 6.4 ×10^−5^) were found at markedly higher densities in the high-risk sample. Notably, PD1 and FOXP3 expression were absent in the low-risk sample across all ROIs. This pattern suggests that a T-cell-inflamed yet actively suppressed and functionally exhausted immune microenvironment drives high-risk EMC.

## Discussion

This study presents, to our knowledge, one of the first TempO-Seq whole-transcriptome targeted RNA sequencing analyses of FFPE-derived EMC specimens, combining differential expression profiling, LASSO–Cox prognostic modeling, *in silico* immune deconvolution, and multiplex immunofluorescence validation in a single integrated framework. Despite the unavoidably small sample size of *n* = 12, a direct consequence of this tumor’s extreme rarity, our findings provide biologically plausible candidate biomarkers and a novel characterization of the EMC immune microenvironment. These findings form a hypothesis-generating platform for future prospective validation.

### Prognostic Gene Signature: *PXN, TYMS,* and *H1FX*

LASSO–Cox regression identified *PXN, TYMS,* and *H1FX* as a candidate prognostic gene set from 14 univariate-significant candidates. Given the small sample size of our cohort, this signature is intended to be an exploratory, hypothesis-generating model rather than a clinically applicable tool at this stage. Higher expression of all three genes was associated with poorer overall survival. This finding is consistent with their known oncogenic roles in other malignancies (Chamizo et al., 2015; Chen et al., 2021; Lee et al., 2014; Liu et al., 2024; Meng, Zhang & Xu, 2023).

A notable multi-omic finding in this study is the identification of *PXN* as a candidate prognostic biomarker in EMC. While we must be mindful of potential circularity when selecting and evaluating biomarkers within the same limited dataset, *PXN* (paxillin) was supported across multiple analytical layers, including differential expression analysis, LASSO-based feature selection, and protein-level validation by multiplex immunofluorescence (*P* = 0.008), making it the only marker in this study with concordant transcriptomic and spatial protein evidence.

*PXN* is a focal adhesion scaffold protein that coordinates integrin signaling, cytoskeletal reorganization, and cell migration. Elevated *PXN* expression has been consistently associated with tumor invasion, metastasis, and poor prognosis across multiple cancer types, including hepatocellular carcinoma, ovarian cancer, and non-small cell lung cancer (Chen et al., 2021; Liu et al., 2023; Noh et al., 2021). In our cohort, *PXN* enrichment in mitotic spindle and cell motility pathways further supports its role in driving aggressive tumor behavior. Collectively, these findings position *PXN* as a biologically plausible and preliminarily supported marker of poor prognosis in EMC, warranting essential validation in larger, independent cohorts. *TYMS* (Thymidylate Synthase) catalyzes the reductive methylation of deoxyuridine monophosphate to thymidine monophosphate, a rate-limiting step in de novo DNA synthesis. Its overexpression is a well-established mechanism of resistance to fluoropyrimidine- and antifolate-based chemotherapies. It is also associated with aggressive tumor biology and poor prognosis in colorectal, lung, hepatocellular, and gastroenteropancreatic neuroendocrine tumors (Chamizo et al., 2015; Geng et al., 2024; Li et al., 2021; Pei et al., 2026; Zhang et al., 2020). In the sarcoma field, *TYMS* has been identified as a prognostic biomarker in retroperitoneal liposarcoma and uterine leiomyosarcoma (Rakic et al., 2023). Our finding of elevated *TYMS* in poor-prognosis EMC extends this association to an additional sarcoma subtype. While external cross-cohort analysis (GSE24369 and GSE6481) is consistent with the baseline *TYMS* expression in EMC, which is broadly comparable to other sarcomas, its distinct upregulation within the poor-prognosis subset of EMC suggests a potential role as an intra-tumoral risk stratification marker, pending external confirmation to rule out model overfitting. While *TYMS* is a known determinant of response to thymidylate synthase-targeting agents in other cancers, its clinical relevance in EMC remains untested; therefore, any therapeutic implications should be considered hypothesis-generating.

*H1FX* (H1 histone family member X) is a member of the linker histone H1 family, which regulates higher-order chromatin compaction and global gene expression. Dysregulation of H1 variants has been linked to altered chromatin accessibility, epithelial-to-mesenchymal transition (EMT), and cancer progression (Fyodorov et al., 2018; Izzo & Schneider, 2016; Saha & Dalal, 2021; Wei, Xu & Lau, 2020). Notably, in our cross-study validation, *H1FX* was significantly upregulated in EMC compared with other chondrosarcomas (GSE6481, *P* = 0.001). This finding suggests that *H1FX* acts not only as a prognostic driver within EMC but also as a distinct molecular feature separating EMC from other cartilaginous tumors. Notably, H1FX protein was not detected by immunofluorescence in either specimen. This represents a negative finding that weakens its immediate translational potential as a protein biomarker. While this may reflect technical limitations of detecting chromatin-associated proteins in FFPE tissue, it also raises the possibility that *H1FX* is not reliably expressed at the protein level in EMC. Alternative validation modalities, such as proximity ligation assay or mass spectrometry-based proteomics, may be required to confirm H1FX protein expression in future studies. Despite this limitation, the strong RNA-level prognostic signal reflected by its LASSO–Cox coefficient of 1.58 suggests that *H1FX* carries the greatest penalized contribution to the risk score within this specific dataset. However, such a high coefficient derived from a small cohort must be interpreted with caution due to the inherent susceptibility to model overfitting. Combined with its significant lineage-specific upregulation in EMC, these data support further exploratory investigation of *H1FX* as both a potential epigenetic hallmark and candidate prognostic driver of this tumor subtype, provided future studies can independently validate its role and overcome current sample size limitations.

### Model performance and the challenge of ultra-small cohort prognostics

The bootstrap-corrected C-index of 0.10 compared with an apparent C-index of 1.00 supports substantial model overfitting. This finding is mathematically expected and does not invalidate the biological signal. Rather, it reflects the fundamental statistical challenge of constructing a multivariable model from a dataset with *n* = 12 and only five events. The combination of LASSO regularization, LOOCV model selection, and 1,000-resample bootstrap optimism correction represents the methodological best practice for this cohort size. No additional within-study approach can substitute for an independent external validation cohort. We therefore explicitly frame the three-gene risk score as an exploratory hypothesis-generating tool and caution against direct clinical application without prospective validation.

The statistical tools applied here, including the identification of *PXN, TYMS,* and *H1FX* as individually prognostic by univariate Cox regression, are more robust than the combined multivariable model. These individual associations are less susceptible to the overfitting that afflicts the composite risk score. The transcriptomic associations of individual genes with survival, supported by concordant protein and cross-study data where available, represent the most reliable findings of this study.

### Immune microenvironment heterogeneity in EMC

A central and novel finding of this study is the characterization of distinct immune microenvironments in high-risk and low-risk EMC tumors, suggesting that EMC may exhibit greater immune heterogeneity than is typically appreciated in soft-tissue sarcomas. By integrating bulk transcriptomic deconvolution with spatially resolved mIF, we identified two complementary immunological phenomena. The first was a potentially protective B-cell-enriched phenotype in low-risk tumors, whereas the second was a spatially restricted exhausted T-cell and regulatory T-cell microenvironment in high-risk tumors.

*In silico* immune deconvolution using a 6-algorithm consensus approach revealed that low-risk tumors are characterized by greater global B-cell infiltration, a finding supported by multiple independent algorithms (TIMER, CIBERSORT-ABS, and MCP-counter). This is biologically plausible and consistent with prior studies demonstrating a favorable prognostic role for tumor-infiltrating B cells and tertiary lymphoid structures (TLS) in multiple cancer types, including sarcomas (Petitprez et al., 2020). B cells promote anti-tumor immunity through antibody production, antigen presentation, and the activation of cytotoxic T-cell responses. The global enrichment of B cells in low-risk EMC tumors aligns with this broader literature and is supported by our gene set enrichment analysis (GSEA), which identified upregulation of adaptive immune pathways in good-prognosis tumors.

Despite this consistent transcriptomic signal, the mIF analysis did not demonstrate a significant difference in B-cell density between the representative specimens. This discrepancy likely reflects fundamental differences in measurement scale and spatial resolution. Bulk RNA sequencing-based deconvolution captures global immune composition across the entire tumor, including spatially organized structures such as tertiary lymphoid structures, whereas mIF evaluates limited, localized regions of interest and may miss spatially restricted immune aggregates. In addition, the mIF analysis was performed on only two representative cases, further limiting its ability to reflect cohort-level trends. Therefore, these findings should be interpreted as complementary rather than contradictory.

The mIF analysis provided critical spatial and functional context to the high-risk microenvironment. While bulk transcriptomic deconvolution of broad T-cell lineages (total CD4^+^ and CD8^+^) showed little variation between groups, high-resolution mIF of the representative high-risk specimen revealed significantly elevated CD3^+^CD8^+^PD1^+^ exhausted cytotoxic T cells (*P* = 6.4 × 10^−5^), CD3^+^CD4^+^PD1^+^ exhausted helper T cells (*P* = 0.006), and FOXP3^+^ regulatory T cells (*P* = 0.006) compared with the low-risk specimen. These findings are derived from two individual cases and must be interpreted as descriptive and hypothesis-generating, not as cohort-level evidence. This discrepancy illustrates the well-documented limitations of bulk RNA-seq deconvolution, which frequently struggles to resolve closely related, rare functional subsets (such as PD1^+^ *vs.* PD1^−^ T cells) due to transcriptomic spillover (Finotello & Trajanoski, 2018; Sturm et al., 2019).

The mIF immune profile characterizes high-risk EMC as a T-cell-inflamed but functionally exhausted microenvironment. Tumor-infiltrating T cells are recruited but actively suppressed through inhibitory signaling pathways (PD1) and immunoregulatory mechanisms (Tregs). The profound enrichment of Tregs reinforces this immunosuppressive state, as Treg cells actively suppress cytotoxic T-cell activity and promote immune tolerance (Basurto-Olvera, Serrano & Maldonado-Bernal, 2025; Kim, Kim & Lee, 2020; Wang et al., 2024). Similar immune evasion phenotypes have been associated with adverse outcomes and resistance to therapy across multiple malignancies (Barnett et al., 2010; Parra et al., 2023; Shou et al., 2016).

The GSEA pathway results provide a transcriptome-wide mechanistic framework that is biologically coherent with the immune cell infiltration findings from TIMER. The significant enrichment of IL2–STAT5 signaling in good-prognosis tumors (*P* < 0.005) is particularly noteworthy in the context of immune evasion. Although IL-2/STAT5 signaling is essential for the maintenance of cytotoxic CD8^+^ T cells, it is also the dominant survival and proliferative signal for FOXP3^+^ Tregs, which are cells that actively suppress effector immunity and promote immune tolerance (Li et al., 2020a; Shan et al., 2022; Togashi, Shitara & Nishikawa, 2019). The spatial finding of significantly elevated Tregs in the high-risk specimen (*P* = 0.006) is directionally consistent with the enrichment of IL2–STAT5 signaling observed in good-prognosis tumors by GSEA, suggesting that IL2–STAT5-driven Treg expansion may contribute to the immunosuppressive microenvironment of high-risk EMC. This interpretation must be framed as hypothesis-generating, given the *n* = 2 mIF specimens and the GSEA thresholds applied.

Conversely, the enrichment of allograft rejection, complement, TNF-α signaling *via* NF-κB, and interferon-γ response pathways in good-prognosis tumors, while not individually reaching conventional significance, collectively supports the hypothesis that active adaptive immune engagement is a factor in favorable disease outcomes in EMC. The allograft rejection hallmark gene set, in particular, is driven by genes encoding cytotoxic effector molecules, MHC class I/II antigens, and T cell activation markers. Its enrichment in good-prognosis cases is consistent with an immunologically active microenvironment capable of mounting effective anti-tumor responses. These GSEA observations are directionally aligned with the greater B cell and CD8^+^ T cell estimates identified by TIMER in the low-risk group. They are also consistent with the broader literature demonstrating that tertiary lymphoid structures and B cell infiltration predict favorable outcomes in soft-tissue sarcomas (Fridman et al., 2023; Laumont et al., 2022; Ma et al., 2024).

### Multi-dimensional utility of the three-gene candidate

The cross-cohort evaluation of our three-gene candidate (*PXN, TYMS, H1FX*) across three independent GEO cohorts refines the biological interpretation of these candidate biomarkers and supports their potential clinical value. Rather than functioning solely as general prognostic markers, this signature appears to capture complementary dimensions of tumor biology that may inform future, larger-scale risk stratification efforts.

The cross-cohort analysis highlights the dual nature of our gene panel. In the GSE24369 dataset, the expression of all three genes in EMC was not statistically distinguishable from that in other soft-tissue sarcomas. However, when compared against cartilaginous tumors in the GSE6481 dataset, EMC exhibited a distinct transcriptional profile, most notably the significant upregulation of *H1FX* (*P* = 0.001). This aligns with the contemporary understanding that, despite its historical nomenclature, EMC is a distinct soft tissue entity with no true cartilaginous differentiation. In this context, *H1FX* may act as a lineage-enriched epigenetic anchor, distinguishing EMC from true cartilaginous tumors while simultaneously associating with poor prognosis within our cohort.

Conversely, *PXN* and *TYMS* showed stable baseline expression across the broad spectrum of soft-tissue sarcomas (GSE24369) and lower expression relative to classic chondrosarcomas (GSE6481). Within the EMC microenvironment, however, the upregulation of these two genes potentially demarcates highly aggressive, poorly responding tumors. Therefore, *PXN* and *TYMS* may serve as candidate indicators of high-risk intra-tumoral behavior rather than lineage definers.

By integrating a lineage-specific feature (*H1FX*) with core drivers of tumor progression (*PXN* and *TYMS*), this three-gene panel provides a candidate multi-dimensional tool for future investigation. It is reliably detectable across independent technologies (microarray *vs.* TempO-Seq *vs.* MI-ONCOSEQ) and platforms (FFPE *vs.* fresh-frozen tissue), establishing a baseline foundation for future prospective validation.

However, these findings must be interpreted cautiously due to several inherent limitations in cross-cohort comparisons. Most notably, the lack of annotated survival data in these external datasets precludes the direct validation of prognostic outcomes, rendering our model strictly hypothesis-generating at this stage. Furthermore, comparisons between our internal data and the Davis et al. cohort carry methodological constraints, including fundamental differences in library preparation and sequencing depth. Demographic and clinical discrepancies are also present: our internal cohort features a mixed-sex, predominantly Asian population analyzed at the primary tumor site, whereas the external cohort consists entirely of Caucasian males (*n* = 6) biopsied at secondary metastatic sites (*e.g.*, lung, gluteus, thigh). Because tumor microenvironments at metastatic sites often possess distinct transcriptomic profiles compared with primary tumors, future studies utilizing larger, uniformly matched clinical cohorts are required to definitively validate the clinical utility of this signature.

### Future directions and potential clinical relevance

These exploratory findings carry several potential clinical implications, pending prospective validation. First, *PXN* protein expression, if confirmed in larger independent cohorts, could eventually serve as a routine immunohistochemical marker for EMC risk stratification alongside standard pathological assessment. Second, the identification of a T-cell-exhausted, Treg-infiltrated microenvironment in high-risk tumors raises the possibility of exploring immune checkpoint inhibitor (ICI) therapy in this subset of patients. While anti-PD-1/PD-L1 agents have shown activity in a subset of sarcoma patients, the PD1 co-expression identified in high-risk EMC provides a tissue-level target that warrants rigorous evaluation in future preclinical and clinical studies. Third, *TYMS* overexpression highlights a potential therapeutic vulnerability, as agents that target thymidylate synthase (such as 5-fluorouracil or pemetrexed) may have differential efficacy based on *TYMS* expression level. However, using *TYMS* to prospectively guide chemotherapy selection in EMC remains strictly theoretical at this stage and requires dedicated investigation. Fourth, functional validation using EMC cell line models such as H-EMC-SS should be pursued to evaluate whether *PXN, TYMS,* and *H1FX* directly regulate invasive behavior, proliferation, or chemotherapy resistance in EMC. Knockdown or overexpression studies targeting these genes *in vitro*, followed by *in vivo* xenograft validation, would provide mechanistic evidence to complement the transcriptomic and protein-level associations reported here.

### Limitations

Several limitations of this study should be acknowledged. The most significant limitation is the extremely small cohort size (*n* = 12), reflecting the rarity of EMC and inherently limiting statistical power. Consequently, the findings should be interpreted with caution and require external validation in independent cohorts before any clinical application can be considered. The retrospective design and variability in FFPE block age spanning approximately two decades may introduce pre-analytical confounding. Although this issue was partially mitigated by the use of TempO-Seq, which is optimized for archival specimens, together with stringent quality control thresholds, residual technical variability cannot be entirely excluded. In addition, statistical power estimates for RNA sequencing analyses rely on assumptions regarding biological replicates and dispersion parameters. Given the ultra-rare nature of EMC and the limited sample size available, these estimates should therefore be interpreted as approximate indicators rather than definitive measures of statistical power.

The dichotomization of patients at 8 years was chosen to create balanced groups (*n* = 6 per group) based on clinically meaningful follow-up intervals. However, this threshold should not be interpreted as a universal prognostic cutoff. In addition, *H1FX* protein was not detectable by immunofluorescence, preventing validation of this candidate biomarker at the protein level. Although the mIF panel enabled detailed characterization of immune cell phenotypes, it represents only a single temporal snapshot of the tumor microenvironment. It therefore cannot capture the dynamic evolution of immune responses throughout disease progression. Furthermore, functional validation of *PXN, TYMS,* and *H1FX* was not performed in this study. Evaluating the expression and functional effects of these proteins, including their impact on cell invasiveness, proliferation, and chemotherapy sensitivity, using available EMC cell line models such as H-EMC-SS, represents an important priority for future work. Such studies will be necessary to establish whether these transcriptomic associations reflect direct mechanistic drivers of aggressive tumor biology or are markers of broader oncogenic programs.

Another limitation concerns the cross-study comparison using the dataset GSE24369, which was originally designed for diagnostic rather than prognostic analysis and includes only a small number of EMC samples (*n* = 6). This restricts the ability to perform robust external validation using publicly available data.

An additional methodological consideration relates to the gene selection strategy used for prognostic modeling. Differential expression analysis was initially conducted between clinically defined prognosis groups, after which candidate genes were evaluated using Cox proportional hazards regression and LASSO feature selection. Because differential gene expression was initially defined using prognostic groups derived from survival outcomes, a degree of circularity in biomarker discovery cannot be fully excluded. In small exploratory cohorts, this design may inflate the apparent association between gene expression and clinical outcomes. To mitigate this potential bias, we applied several safeguards, including strong penalization through LASSO regularization, leave-one-out cross-validation during model training, and bootstrap-based optimism correction of model performance estimates. Because the prognostic model was developed from a small cohort with limited survival events, the stability of selected predictors may be sensitive to sample variability despite the use of penalized regression and cross-validation. Nevertheless, given the limited sample size, the resulting gene signature should be regarded as hypothesis-generating rather than definitive. Independent validation in larger, multicenter EMC cohorts will be required to confirm the prognostic relevance and generalizability of these candidate biomarkers.

## Conclusions

This study demonstrates that transcriptomic profiling of archival FFPE tissue is feasible in ultra-rare sarcomas such as EMC and can yield biologically informative signals. *PXN, TYMS,* and *H1FX* were identified as candidate prognostic biomarkers from an integrated RNA sequencing, immunofluorescence, and cross-study analysis, with *PXN* showing the most robust multi-level support. The immune microenvironment of EMC appears heterogeneous rather than immunologically inert, with a B-cell-enriched phenotype associated with a favorable prognosis and a spatially localized exhausted T-cell and regulatory T-cell profile observed in high-risk disease. These findings provide a foundation for future investigation of immune biology and potential therapeutic strategies in EMC. However, given the limited sample size and exploratory design, all results should be interpreted as hypothesis-generating and require validation in larger, multi-center cohorts before clinical translation.

## Supplemental Information

10.7717/peerj.21497/supp-1 Table S1 Univariate Cox proportional hazards regression analysis of clinical variables in patients with extraskeletal myxoid chondrosarcoma (*n* = 12; events = 5)

10.7717/peerj.21497/supp-2 Table S2 Univariate Cox proportional hazards regression analysis and permutation test Univariate Cox proportional hazards regression analysis was performed to identify genes associated with patient survival. Empirical *P* values were obtained through permutation testing (10,000 permutations) to assess the statistical significance of individual gene associations. Abbreviations: CI, confidence interval; Coef., coefficient; CoxPH, Cox proportional hazards; HR, hazard ratio.

10.7717/peerj.21497/supp-3 Figure S1 PCA plots and heatmap plots for before (A, B) and after (C, D) corrected batch effect of the gene expression with an outlier (A) PCA plot displaying the gene expression profiles before correcting for batch effects. The *x*-axis represents principal component 1 (PC1), accounting for 53.57% of the variance, and the *y*-axis represents principal component 2 (PC2), accounting for 24.57% of the variance. Each dot represents a patient, with green dots indicating patients with new tissue blocks and orange dots representing those with old tissue blocks. The density plots along the *x* and *y* axes show the distribution of data points for PC1 and PC2. A green circle highlights an outlier. (B) Heatmap showing normalized gene expression levels before batch correction. Patients (columns) are clustered based on their gene expression profiles, with rows representing individual genes. Patients are color-coded according to the age of the tissue block (new: green, old: orange). Red indicates higher gene expression, while blue represents lower expression. (C) PCA plot after correcting for batch effects, with PC1 and PC2 now accounting for 32.57% and 27.03% of the variance, respectively. As in Fig. S1A, green and orange dots indicate patients with new and old tissue blocks, respectively. A green circle highlights an outlier. The density plots again show the compactness of data points. (D) Heatmap of normalized gene expression levels after batch correction. Similar to Fig. S1B, patients (columns) are clustered based on gene expression profiles, and rows represent individual genes. The heatmap shows gene expression levels across patients, with red indicating higher expression and blue indicating lower expression. **Abbreviations**: EMC, extraskeletal myxoid chondrosarcoma; PCA, principal component analysis.

10.7717/peerj.21497/supp-4 Figure S2 Gene set enrichment analysis (GSEA) of immune-related and biological Hallmark pathways in extraskeletal myxoid chondrosarcoma Lollipop plot showing normalized enrichment score (NES) for selected MSigDB Hallmark pathways, comparing the poor-prognosis (*n* = 6) and good-prognosis (n=6) cohorts. The analysis was performed using the *fgsea* package. Node color indicates the clinical group in which the pathway is enriched: orange indicates enrichment in the poor-prognosis group (positive NES), while blue indicates enrichment in the good-prognosis group (negative NES). The size of each node corresponds to the statistical significance, scaled by −log10(*P* value). Significant enrichment (*P* < 0.005) was observed for the Apoptosis pathway in the poor prognosis cohort and the IL2-STAT5 signaling pathway in the good-prognosis cohort. Other evaluated immune-related pathways (e.g., Interferon Gamma Response, Inflammatory Response) did not reach statistical significance in this cohort. **Abbreviations**: EMC, extraskeletal myxoid chondrosarcoma; GSEA, gene set enrichment analysis; MSigDB, Molecular Signatures Database; NES, normalized enrichment score.

10.7717/peerj.21497/supp-5 Figure S3 Construction of the three-gene prognostic signature using LASSO Cox regression (A) Coefficient profiles of selected features as a function of the regularization parameter *λ*. (B) Partial likelihood deviance plotted against log(*λ*) using the LASSO–Cox regression model. (C) Kaplan–Meier survival curve stratified by the three-gene (*PXN, TYMS,* and *H1FX*) risk score. **Abbreviations**: EMC, extraskeletal myxoid chondrosarcoma; *H1FX*, H1 histone family member X; LASSO, Least Absolute Shrinkage and Selection Operator; OS, overall survival; *PXN*, paxillin; *TYMS*, thymidylate synthase.

10.7717/peerj.21497/supp-6 Figure S4 Comparative immune cell infiltration analysis using a multi-algorithm ensemble approach in EMC (*n* = 12) Grouped boxplots showing the estimated abundance of six canonical immune lineages (B cells, CD4+ T cells, CD8+ T cells, macrophages, neutrophils, and myeloid dendritic cells) stratified by prognostic risk group (low-risk, *n* = 6; high-risk, *n* = 6). To ensure cross-method robustness and mitigate algorithm-specific biases, immune infiltration was estimated from bulk RNA sequencing data using six orthogonal deconvolution algorithms (TIMER, CIBERSORT, CIBERSORT-ABS, EPIC, MCP-counter, and xCell). Algorithm-specific high-resolution subpopulations were logically aggregated into the six parent lineages to enable standardized cross-algorithm comparisons. Center lines denote the median, boxes represent the interquartile range, and individual patient estimates are overlaid as jittered points. Statistical differences between the risk groups were evaluated using the non-parametric two-sided Wilcoxon rank-sum test. Significance brackets above each plot display the raw *P* value, the rank-biserial correlation coefficient (R) indicating effect size, and the Benjamini–Hochberg false discovery rate (FDR) adjusted *P* value to account for multiple hypothesis testing across the 36 simultaneous comparisons. **Abbreviations**: CIBERSORT, Cell-Type Identification By Estimating Relative Subsets Of RNA Transcripts; CIBERSORT-ABS, CIBERSORT absolute mode; EMC, extraskeletal myxoid chondrosarcoma; EPIC, Estimating the Proportions of Immune and Cancer cells; FDR, false discovery rate; MCP-counter, Microenvironment Cell Populations-counter; TIMER, Tumor Immune Estimation Resource; xCell, a computational method for cell-type enrichment analysis.

10.7717/peerj.21497/supp-7 Figure S5 Bootstrap calibration plots at 1–5 years demonstrate polarized predicted survival probabilities consistent with model overfitting in this small cohort (*n* = 12) The directional separation between low-risk (blue) and high-risk (red) groups is consistent across all time points, supporting the biological signal of the three-gene signatures.

10.7717/peerj.21497/supp-8 Figure S6 Integrated exploratory evaluation of the three-gene expression panel in extraskeletal myxoid chondrosarcoma. (A–B) Log2-normalized microarray expression intensities of the candidate genes (*PXN, TYMS,* and *H1FX*) across 25 independent EMC tumors sourced from two distinct publicly available cohorts (GSE24369, *n* = 6; GSE6481, *n* = 19). Horizontal black bars denote the median expression level, with error bars representing the interquartile range. The candidate genes show consistent baseline detection across these independent external cohorts, comparable to the levels observed in the primary study. (C) Comparison of normalized expression (Z-scores) between the primary study cohort (*n* = 12, TempO-Seq) and the external MI-ONCOSEQ dataset (*n* = 6, Davis et al., 2017). Statistical analysis indicates no significant differences in the expression levels of *PXN, H1FX*, and *TYMS* between platforms (Mann–Whitney U test, all *P* > 0.8), supporting the consistency of these transcriptomic markers across disparate sequencing technologies. **Abbreviations**: EMC, extraskeletal myxoid chondrosarcoma; *H1FX*, H1 histone family member X; *PXN*, paxillin; TempO-Seq, Templated Oligo-Sequencing; *TYMS*, thymidylate synthase.

10.7717/peerj.21497/supp-9 Data S1 Raw data

## Additional Information and Declarations

### DNA Deposition

The following information was supplied regarding the deposition of DNA sequences: The raw sequence data are available at NCBI BioProject: PRJNA1357027.

### Data Availability

The following information was supplied regarding data availability: The data is available in the Supplemental Files. The raw sequence data are available at NCBI BioProject: PRJNA1357027.
