# Supplementary material for: Prognostic biomarkers for enhanced risk stratification in extraskeletal myxoid chondrosarcoma: a retrospective cohort study
Source: PeerJ. 2026 Jul 13;14:e21497. doi: 10.7717/peerj.21497 (PMC13374579; doi:10.7717/peerj.21497)
Supplement: Table S1 [file peerj-14-21497-s001.docx]

| **Variable** | **HR** | **95% CI** | ***P* value** | **Method** |
| --- | --- | --- | --- | --- |
| Age | 0.97 | 0.88 - 1.07 | 0.53 | Standard Cox |
| Ethnicity | 2.31 | 0.25 - 21.17 | 0.459 | Standard Cox |
| FISH | 0.84 | 0.14 - 5.19 | 0.85 | Standard Cox |
| Size | 0.99 | 0.87 - 1.12 | 0.824 | Standard Cox |
| Cellularity | 10.23 | 0.88 - 119.42 | 0.064 | Standard Cox |
| Location | 2.31 | 0.35 - 15.49 | 0.387 | Standard Cox |
| Block age | 0.79 | 0.13 - 4.85 | 0.799 | Standard Cox |
| Metastasis | 5.28 | 1.71 - Inf | 0.179 | Firth's Penalized |
| Margin status | 14.13 | 4.06 - Inf | 0.021 | Firth's Penalized |
