# Supplementary material for: Prognostic biomarkers for enhanced risk stratification in extraskeletal myxoid chondrosarcoma: a retrospective cohort study
Source: PeerJ. 2026 Jul 13;14:e21497. doi: 10.7717/peerj.21497 (PMC13374579; doi:10.7717/peerj.21497)
Supplement: Table S2 — Univariate Cox proportional hazards regression analysis was performed to identify genes associated with patient survival. Empirical P values were obtained through permutation testing (10,000 permutations) to assess the statistical significance of individual gene associations. Abbreviations: CI, confidence interval; Coef., coefficient; CoxPH, Cox proportional hazards; HR, hazard ratio. [file peerj-14-21497-s002.docx]

| **Gene** | **Coef.** | **HR (95% CI)** | ***P* value** | **Empirical *P* value** |
| --- | --- | --- | --- | --- |
| PXN  TYMS  ZSCAN21  RPTN  MCM2  TRIM11  PXDN  NUF2  COL16A1  CD300E  IFT2  H1FX  PTCH2  CCDC97 | 0.7944  1.286  1.496  0.3483  0.6404  1.05  1.185  0.6458  -1.674  0.6172  0.4461  0.8175  0.5526  0.5938 | 2.213(1.181-4.148)  3.617(1.201-10.89)  4.463(1.194-16.68)  1.417(1.021-1.966)  1.897(1.038-3.467)  2.859(1.055-7.746)  3.271(1.059-10.11)  1.907(1.023-3.557)  0.1875(0.036-0.954)  1.854(1.014-3.388)  1.562(1.01-2.416)  2.265(1.017-5.043)  1.738(1.006-3.002)  1.811(1.006-3.258) | 0.01321  0.0227  0.02617  0.03724  0.03731  0.03887  0.03951  0.04227  0.04377  0.04485  0.04487  0.04535  0.04756  0.0476 | 0.*0*004  0.*0*002  0.*0*004  0.0224  0.0198  0.0224  0.0102  0.0368  0.0082  *0.0412*  *0.0404*  *0.0224*  *0.0264*  *0.0486* |
