# Supplementary figures and images for: Prognostic biomarkers for enhanced risk stratification in extraskeletal myxoid chondrosarcoma: a retrospective cohort study
Source: PeerJ. 2026 Jul 13;14:e21497. doi: 10.7717/peerj.21497 (PMC13374579; doi:10.7717/peerj.21497)

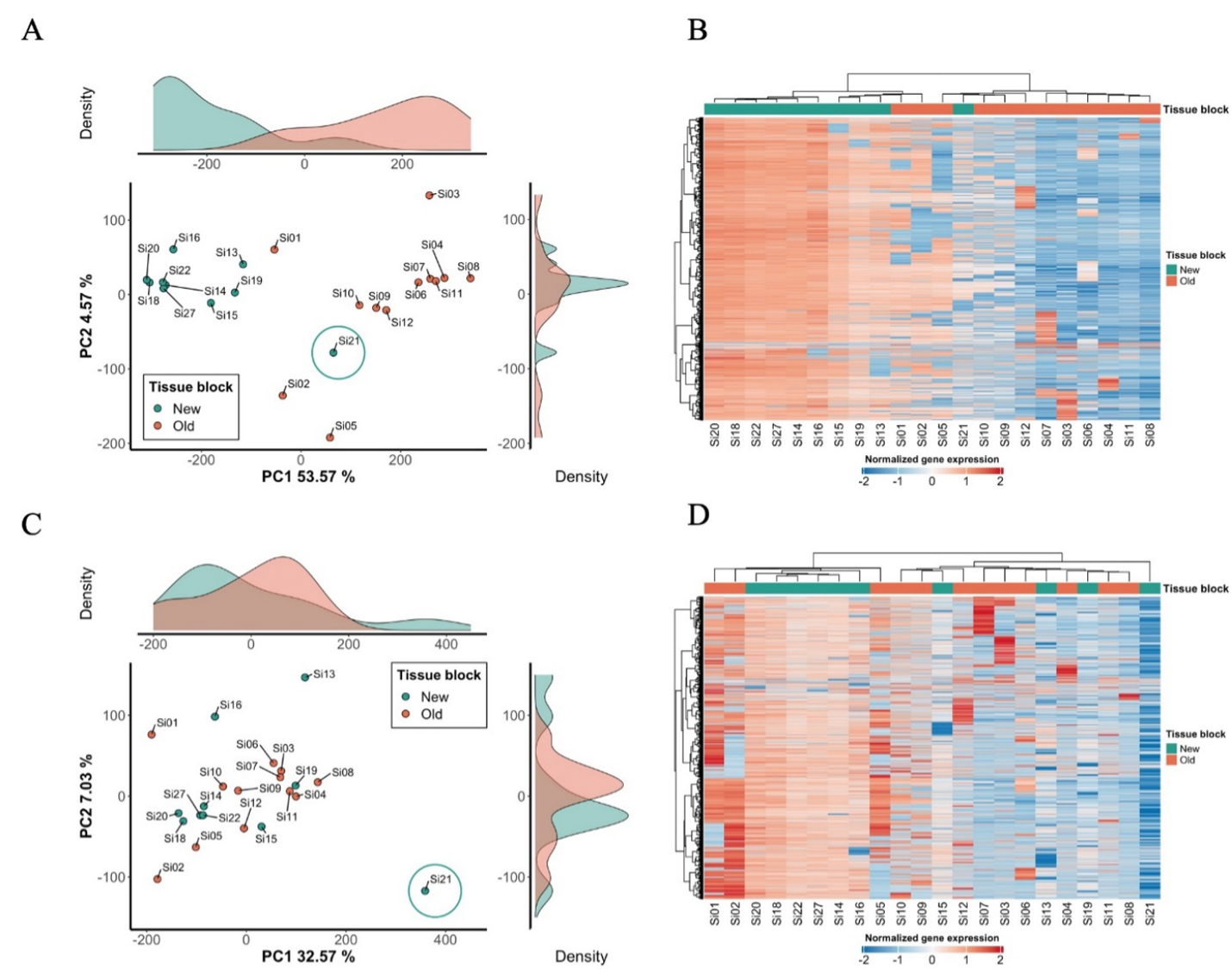

Supplement: Figure S1 — (A) PCA plot displaying the gene expression profiles before correcting for batch effects. The x-axis represents principal component 1 (PC1), accounting for 53.57% of the variance, and the y-axis represents principal component 2 (PC2), accounting for 24.57% of the variance. Each dot represents a patient, with green dots indicating patients with new tissue blocks and orange dots representing those with old tissue blocks. The density plots along the x and y axes show the distribution of data points for PC1 and PC2. A green circle highlights an outlier. (B) Heatmap showing normalized gene expression levels before batch correction. Patients (columns) are clustered based on their gene expression profiles, with rows representing individual genes. Patients are color-coded according to the age of the tissue block (new: green, old: orange). Red indicates higher gene expression, while blue represents lower expression. (C) PCA plot after correcting for batch effects, with PC1 and PC2 now accounting for 32.57% and 27.03% of the variance, respectively. As in Fig. S1A, green and orange dots indicate patients with new and old tissue blocks, respectively. A green circle highlights an outlier. The density plots again show the compactness of data points. (D) Heatmap of normalized gene expression levels after batch correction. Similar to Fig. S1B, patients (columns) are clustered based on gene expression profiles, and rows represent individual genes. The heatmap shows gene expression levels across patients, with red indicating higher expression and blue indicating lower expression. Abbreviations: EMC, extraskeletal myxoid chondrosarcoma; PCA, principal component analysis. [file peerj-14-21497-s003.png]

Immune-related Hallmark Pathways — GSEA

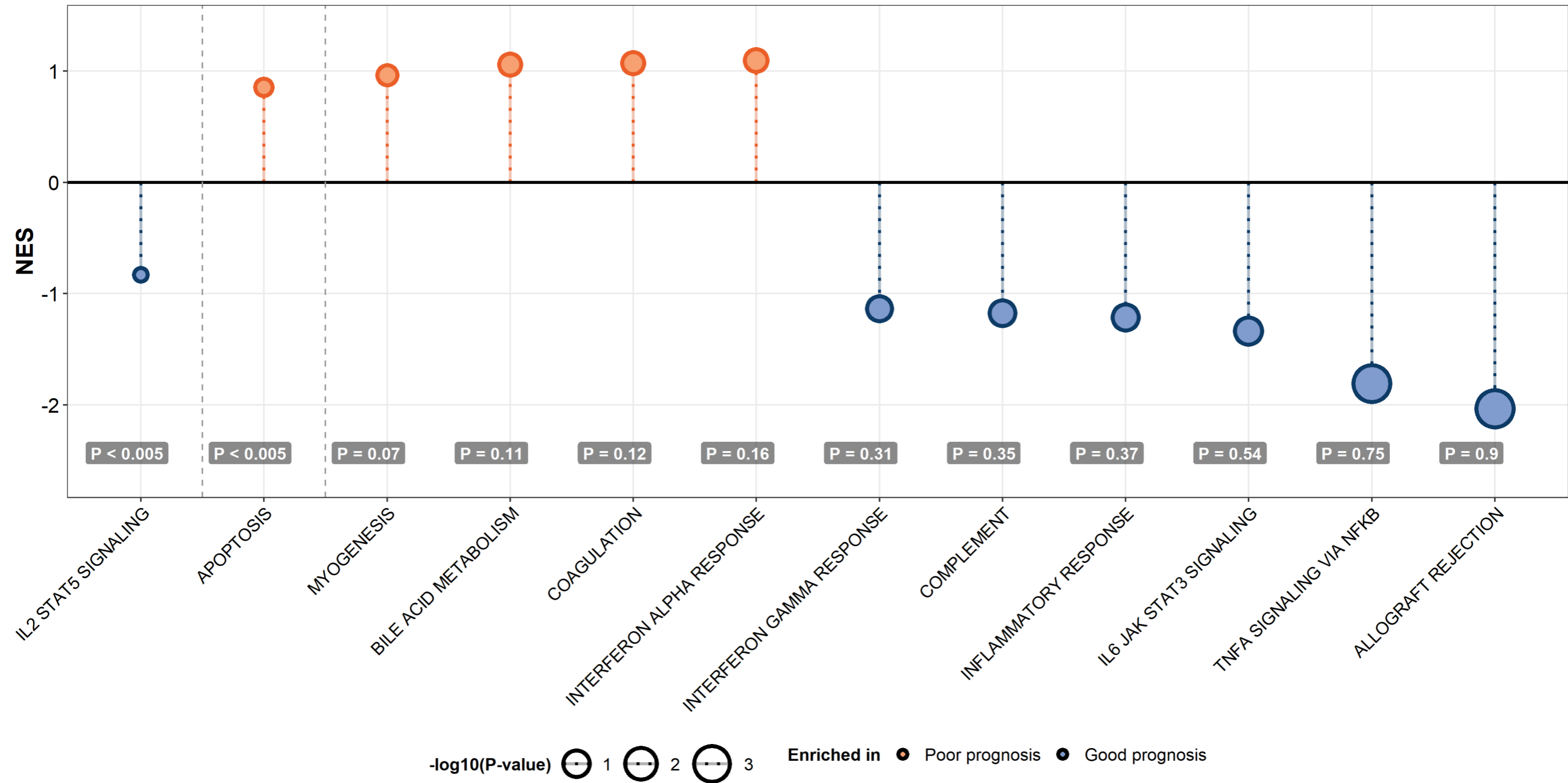

Supplement: Figure S2 — Lollipop plot showing normalized enrichment score (NES) for selected MSigDB Hallmark pathways, comparing the poor-prognosis (n = 6) and good-prognosis (n=6) cohorts. The analysis was performed using the fgsea package. Node color indicates the clinical group in which the pathway is enriched: orange indicates enrichment in the poor-prognosis group (positive NES), while blue indicates enrichment in the good-prognosis group (negative NES). The size of each node corresponds to the statistical significance, scaled by −log10(P value). Significant enrichment (P < 0.005) was observed for the Apoptosis pathway in the poor prognosis cohort and the IL2-STAT5 signaling pathway in the good-prognosis cohort. Other evaluated immune-related pathways (e.g., Interferon Gamma Response, Inflammatory Response) did not reach statistical significance in this cohort. Abbreviations: EMC, extraskeletal myxoid chondrosarcoma; GSEA, gene set enrichment analysis; MSigDB, Molecular Signatures Database; NES, normalized enrichment score. [file peerj-14-21497-s004.pdf]

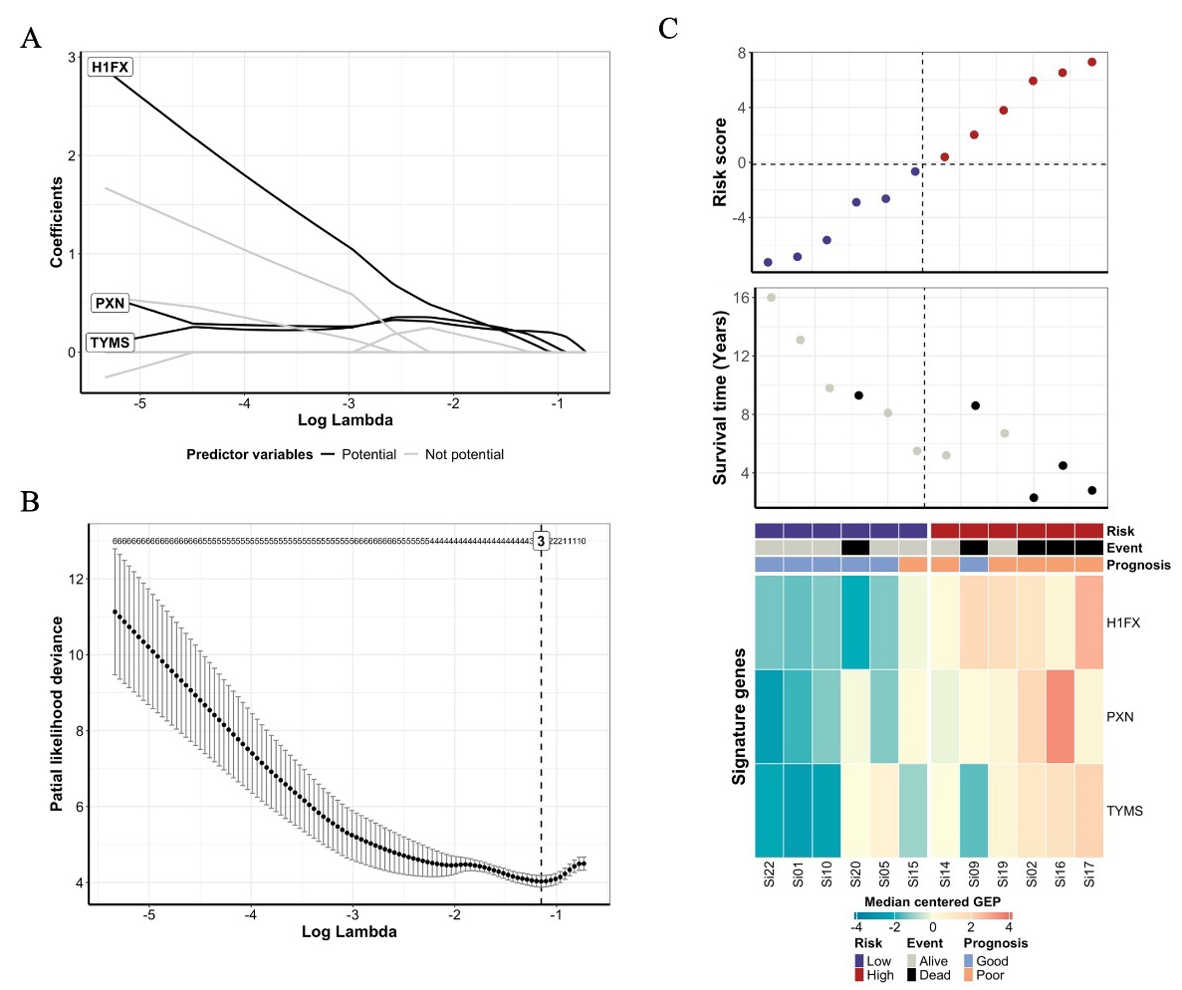

Supplement: Figure S3 — (A) Coefficient profiles of selected features as a function of the regularization parameter λ. (B) Partial likelihood deviance plotted against log(λ) using the LASSO–Cox regression model. (C) Kaplan–Meier survival curve stratified by the three-gene (PXN, TYMS, and H1FX) risk score. Abbreviations: EMC, extraskeletal myxoid chondrosarcoma; H1FX, H1 histone family member X; LASSO, Least Absolute Shrinkage and Selection Operator; OS, overall survival; PXN, paxillin; TYMS, thymidylate synthase. [file peerj-14-21497-s005.png]

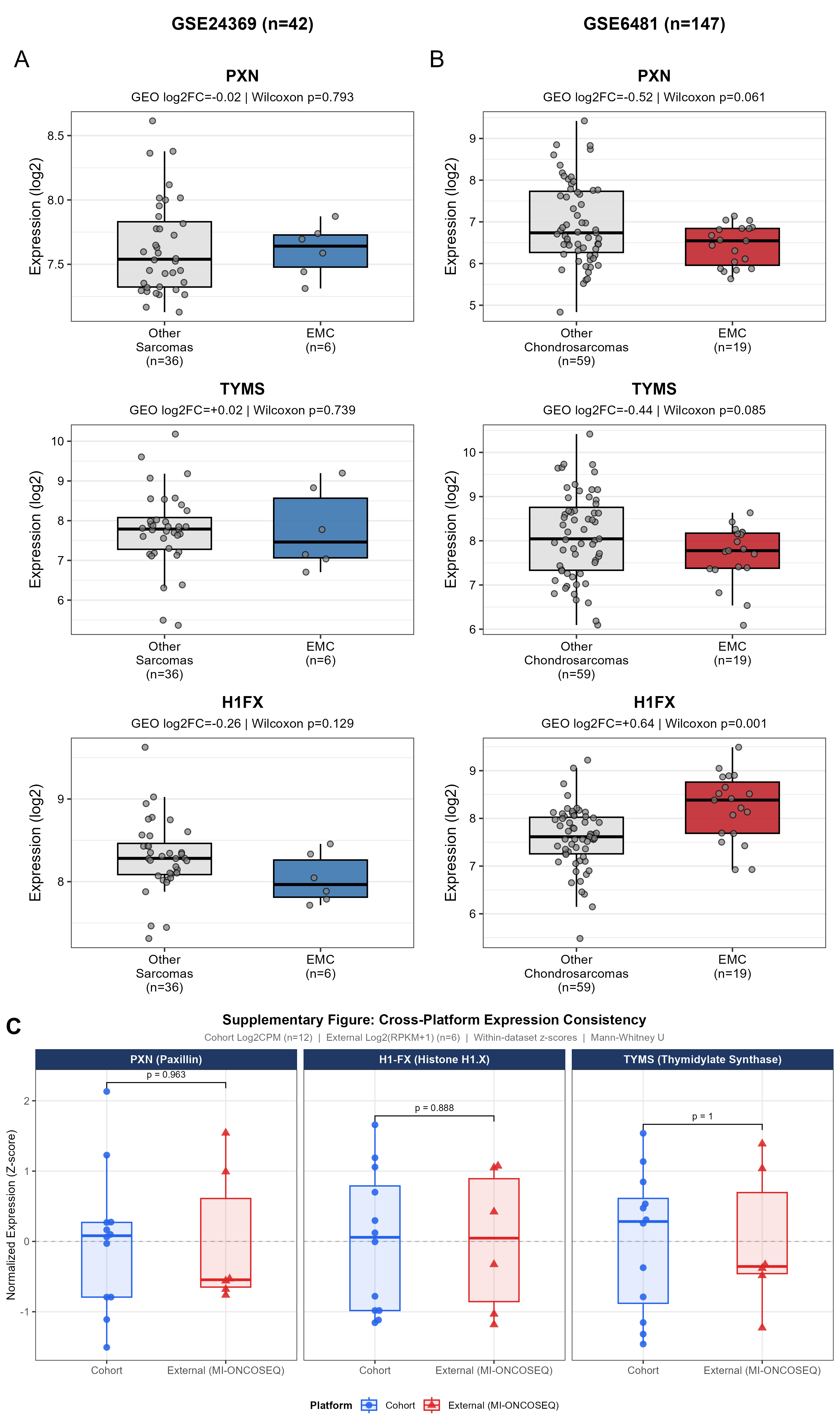

Supplement: Figure S6 — (A–B) Log2-normalized microarray expression intensities of the candidate genes (PXN, TYMS, and H1FX) across 25 independent EMC tumors sourced from two distinct publicly available cohorts (GSE24369, n = 6; GSE6481, n = 19). Horizontal black bars denote the median expression level, with error bars representing the interquartile range. The candidate genes show consistent baseline detection across these independent external cohorts, comparable to the levels observed in the primary study. (C) Comparison of normalized expression (Z-scores) between the primary study cohort (n = 12, TempO-Seq) and the external MI-ONCOSEQ dataset (n = 6, Davis et al., 2017). Statistical analysis indicates no significant differences in the expression levels of PXN, H1FX, and TYMS between platforms (Mann–Whitney U test, all P > 0.8), supporting the consistency of these transcriptomic markers across disparate sequencing technologies. Abbreviations: EMC, extraskeletal myxoid chondrosarcoma; H1FX, H1 histone family member X; PXN, paxillin; TempO-Seq, Templated Oligo-Sequencing; TYMS, thymidylate synthase. [file peerj-14-21497-s008.png]
