# Supplementary material for: Prognostic biomarkers for enhanced risk stratification in extraskeletal myxoid chondrosarcoma: a retrospective cohort study
Source: PeerJ. 2026 Jul 13;14:e21497. doi: 10.7717/peerj.21497 (PMC13374579; doi:10.7717/peerj.21497)

Immune Cell Infiltration by Risk Group — All Deconvolution Methods

Wilcoxon rank-sum test (two-sided); n = 6 per group; FDR correction: Benjamini-Hochberg; effect size: rank-biserial r

Red text = FDR significant (* <0.05, ** <0.01, *** <0.001) | Grey text = not significant (ns)

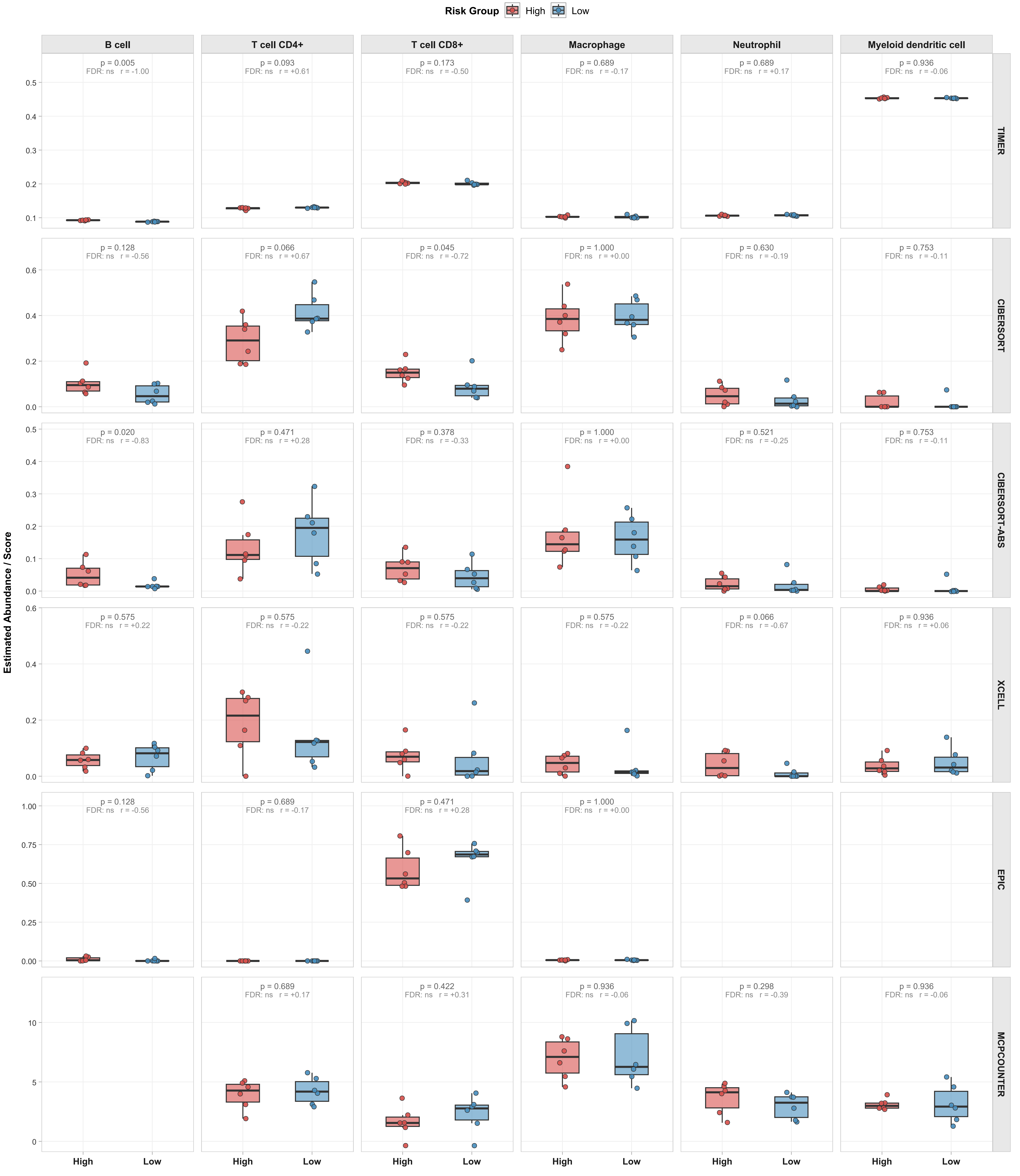

Supplement: Figure S4 — Grouped boxplots showing the estimated abundance of six canonical immune lineages (B cells, CD4+ T cells, CD8+ T cells, macrophages, neutrophils, and myeloid dendritic cells) stratified by prognostic risk group (low-risk, n = 6; high-risk, n = 6). To ensure cross-method robustness and mitigate algorithm-specific biases, immune infiltration was estimated from bulk RNA sequencing data using six orthogonal deconvolution algorithms (TIMER, CIBERSORT, CIBERSORT-ABS, EPIC, MCP-counter, and xCell). Algorithm-specific high-resolution subpopulations were logically aggregated into the six parent lineages to enable standardized cross-algorithm comparisons. Center lines denote the median, boxes represent the interquartile range, and individual patient estimates are overlaid as jittered points. Statistical differences between the risk groups were evaluated using the non-parametric two-sided Wilcoxon rank-sum test. Significance brackets above each plot display the raw P value, the rank-biserial correlation coefficient (R) indicating effect size, and the Benjamini–Hochberg false discovery rate (FDR) adjusted P value to account for multiple hypothesis testing across the 36 simultaneous comparisons. Abbreviations: CIBERSORT, Cell-Type Identification By Estimating Relative Subsets Of RNA Transcripts; CIBERSORT-ABS, CIBERSORT absolute mode; EMC, extraskeletal myxoid chondrosarcoma; EPIC, Estimating the Proportions of Immune and Cancer cells; FDR, false discovery rate; MCP-counter, Microenvironment Cell Populations-counter; TIMER, Tumor Immune Estimation Resource; xCell, a computational method for cell-type enrichment analysis. [file peerj-14-21497-s006.pdf]
