# Supplementary material for: Prognostic biomarkers for enhanced risk stratification in extraskeletal myxoid chondrosarcoma: a retrospective cohort study
Source: PeerJ. 2026 Jul 13;14:e21497. doi: 10.7717/peerj.21497 (PMC13374579; doi:10.7717/peerj.21497)
Supplement: Figure S5 — The directional separation between low-risk (blue) and high-risk (red) groups is consistent across all time points, supporting the biological signal of the three-gene signatures. [file peerj-14-21497-s007.pdf]

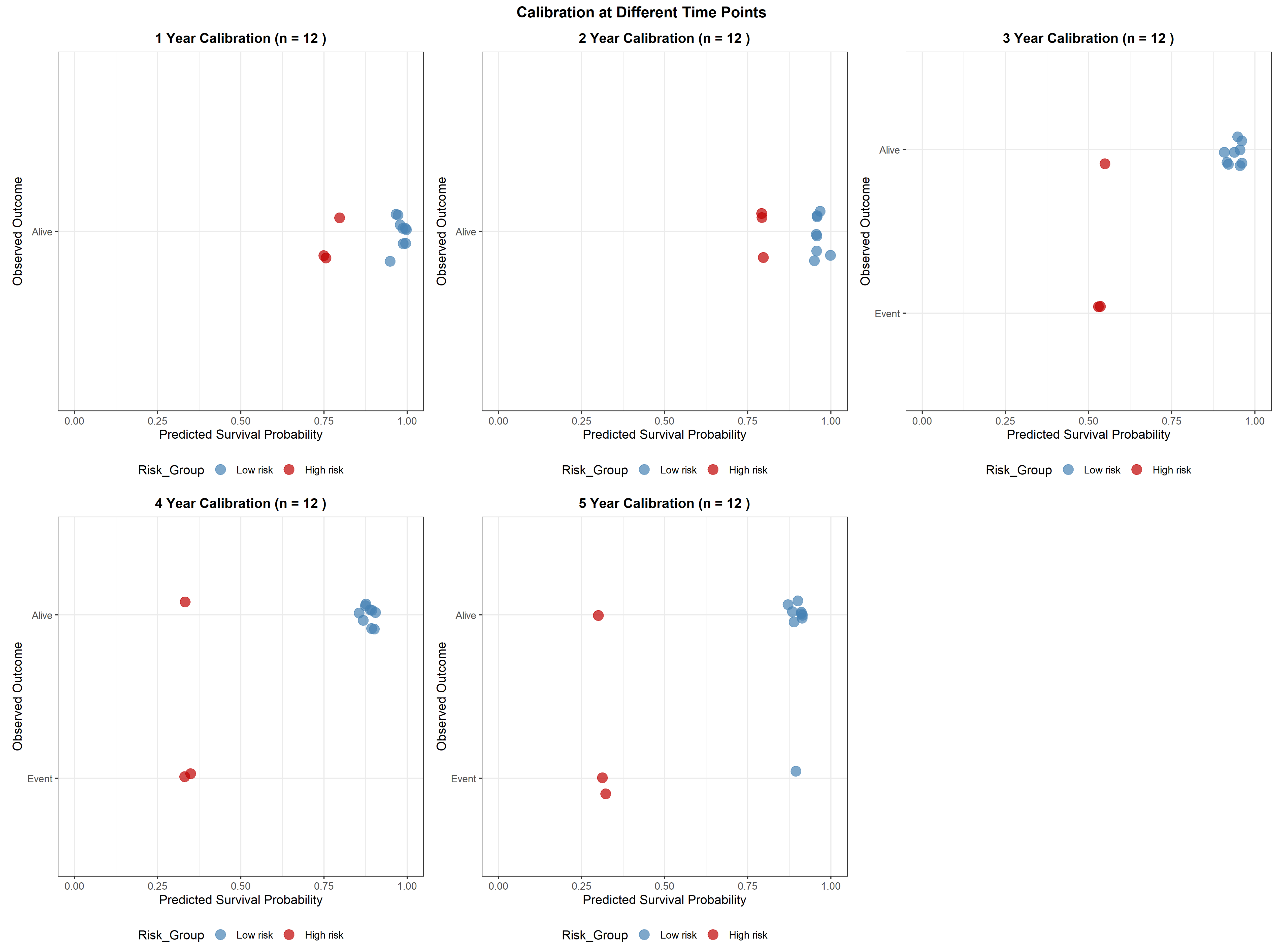
Calibration at Different Time Points
1 Year Calibration (n = 12)
2 Year Calibration (n = 12)
3 Year Calibration (n = 12)
4 Year Calibration (n = 12)
5 Year Calibration (n = 12)
Observed Outcome
Alive
Event
Predicted Survival Probability
0.00
0.25
0.50
0.75
1.00
Risk_Group
Low risk
High risk
